# The non-market benefits of early and partial gains in managing threatened salmon

**DOI:** 10.1371/journal.pone.0220260

**Published:** 2019-08-14

**Authors:** David J. Lewis, Steven J. Dundas, David M. Kling, Daniel K. Lew, Sally D. Hacker

**Affiliations:** 1 Department of Applied Economics, Oregon State University, Corvallis, Oregon, United States of America; 2 Coastal Oregon Marine Experiment Station, Oregon State University, Newport, Oregon, United States of America; 3 Alaska Fisheries Science Center, National Oceanic and Atmospheric Administration, Seattle, Washington, United States of America; 4 Department of Integrative Biology, Oregon State University, Corvallis, Oregon, United States of America; Washington State University, UNITED STATES

## Abstract

Threatened species are increasingly dependent on conservation investments for persistence and recovery. Information that resource managers could use to evaluate investments–such as the public benefits arising from alternative conservation designs–is typically scarce because conservation benefits arise outside of conventional markets. Moreover, existing studies that measure the public benefits of conserving threatened species often do not measure the benefits from partial gains in species abundance that fall short of official recovery, or the benefits from achieving gains in species abundance that happen earlier in time. We report on a stated preference choice experiment designed to quantify the non-market benefits for conservation investments aimed at threatened Pacific Coho salmon (*Oncorhynchus kisutch*) along the Oregon Coast (OC). Our results show that a program aimed at increasing numbers of returning salmon can generate sizable benefits of up to $518 million/y for an extra 100,000 returning fish, even if the species is not officially declared recovered. Moreover, while conservation investment strategies expected to achieve relatively rapid results are likely to have higher up-front costs, our results show that the public attaches substantial additional value of up to $277 million/y for achieving conservation goals quickly. Our results and approach can be used to price natural capital investments that lead to gains in returning salmon, and as inputs to evaluations of the benefits and costs from alternative conservation strategies.

## Introduction

Substantial resources are devoted to recovering threatened species. In the United States (US), the government invested over $1.2 billion annually on species listed under the Endangered Species Act (ESA) from 1989 through 2014 ([1] p. 72). Despite decades of investment, the number of once-threatened species officially considered recovered is small. Only 1% of ESA-listed species have been de-listed [1].

Economists classify the public (or social) benefits of recovering threatened species as primarily non-market, meaning that they cannot be measured from observations of market activity alone [2]. Public benefits may arise from a combination of values that are “use” (e.g., harvest, viewing in the wild, and education) or “non-use” (e.g., existence of the species, future harvest or viewing opportunities, and bequest for future generations) in nature ([2–5]). Prior research that attempts to measure the public’s willingness-to-pay for the recovery of threatened species has focused on valuing a change in official conservation status designations (e.g., threatened vs. recovered under the ESA), with an emphasis on final outcomes at some point in the future (e.g., 30 years from now) [6]. Yet, in reality, few endangered or threatened species have been de-listed, making the value of recovery potentially less important than other values more relevant to ongoing management. For example, it is important to know whether the public places economic value on management that leads to gains in species abundance, but falls short of a recovered designation. In addition, is there any additional public benefit from a recovery trajectory that happens quickly versus slowly?

**Fig 1:**
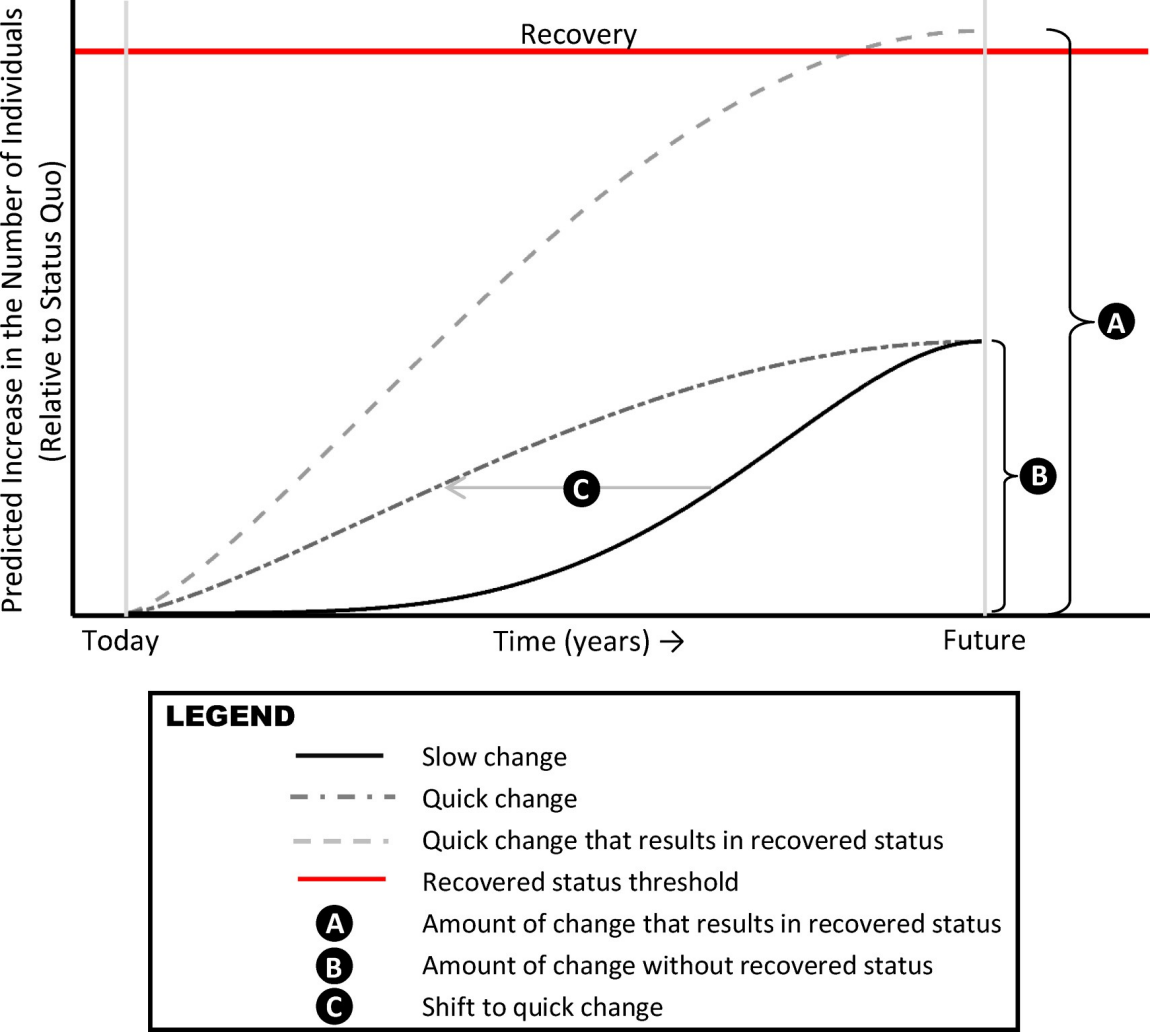
Alternative time paths for increasing the number of individuals of a hypothetical endangered or threatened species.

In this paper, we implement an original choice experiment survey of the general population of residents in the US Pacific Northwest (PNW) and quantify the economic value the public places on various trajectories for increasing the number of returning fish of a threatened salmon species. We focus on Oregon Coast (OC) Coho salmon, a geographically separate group of Pacific Coho salmon (*Oncorhynchus kisutch*) with fish populations that are restricted to OC watersheds and that are listed as threatened under the ESA. Our experimental design allows us to measure the monetary-equivalent public benefit of different attributes of potential future returning OC Coho adult salmon over time. We illustrate these attributes for a generic threatened species in **Fig 1**. As mentioned, prior research has focused on measuring the public benefit of achieving recovered status of a species in the future (Feature A shown in **Fig 1**). We measure the value of such a change for OC Coho salmon, specifically the value of the species being considered officially recovered under the ESA. We also estimate the benefit of different trajectories of *partial* gains in the abundance of returning salmon that fall short of achieving recovered status (Feature B), which allows us to develop a measure of public benefits applicable to the majority of ESA-listed species which have yet to be de-listed. Importantly, because we can value partial gains in returning salmon (Feature B), our econometric model has the flexibility to predict the benefits of a range of potential future outcomes for returning salmon. Another novel feature of our design is its emphasis on the dynamics of a threatened species (Feature C). For a given final number of returning salmon, we can measure how much additional benefit (if any) the public derives from achieving that number of returning fish quickly in time.

Estimating the social benefits the public derives from alternative time paths for increasing the number of individuals of a threatened species is useful in the design and evaluation of policies involving investments in natural capital. The costs of these investments are often measured in monetary units, such as the foregone profits from restricting land use practices to achieve conservation outcomes. Separately quantifying the public’s willingness to pay for natural capital investments in monetary terms–such as what we do in this study–provides the information necessary for decision-makers to weigh the benefits and costs of alternative natural capital investments in the same units [7].

## Methods

We use a stated preference choice experiment survey with a set of realistic recovery scenarios consistent with salmon life histories and management policies to estimate the non-market values for Pacific Coho salmon (*Oncorhynchus kisutch*), specifically within the federally defined Oregon Coast (OC) Coho salmon evolutionary significant unit. Choice experiments consist of survey questions that elicit specific information about preferences [8]. Individuals are asked to choose between two or more conservation alternatives (termed “salmon recovery programs” in our application) that are differentiated by the set of attributes and attribute levels that describe them (e.g., program outcomes and costs). The set of attributes and levels seen by respondents are randomly varied across questions in a survey and across multiple survey versions to maximize the information about underlying preferences that can be derived from an analysis of the choice responses [9].

**Fig 2:**
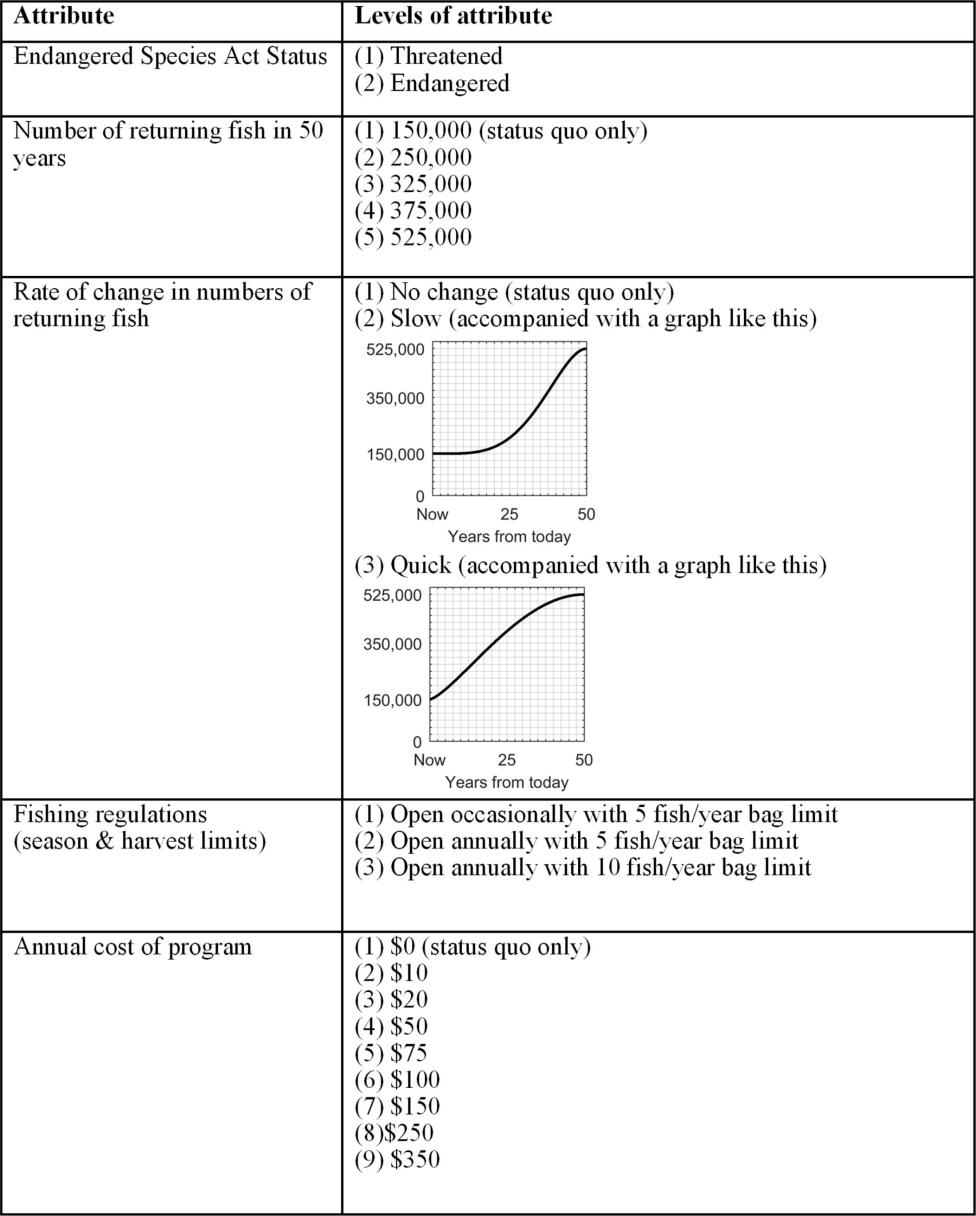
Experimental design attributes and levels of attributes used in the stated preference survey for recovery of Oregon Coast Coho salmon given to US Pacific Northwest residents.

We used two government recovery plans to guide our scenario development for the surveys: the Oregon Coast Coho Conservation Plan for the State of Oregon [10] and the Federal Government's Recovery Plan for Oregon Coast Coho Salmon Evolutionary Significant Unit [11] (*Section 1.a in S1 Text*). While both the State of Oregon’s and the Federal Government’s plans present conservation goals for OC Coho salmon, only the Federal Government plan [11] represents the official opinion of the Federal agency (NOAA) charged with recovering the species. The choice experiment measured preferences for attributes that represent key decision variables for investing in natural capital to help threatened OC Coho salmon (**Fig 2**). An important feature of our experimental design is that for each scenario, we showed survey respondents the number of adult fish that return 50 years from now plus graphical depictions of the rate of increase in the number of returning fish (**Fig 2**). The rate of increase graphs were generated using an application of the Beta function of determinate growth [12]–see *Section 1.a in S1 Text*. The baseline number of returning adult OC Coho salmon is presented to survey respondents as a flat line fixed at 150,000 fish, which is approximately equal to the 22-year annual average of returning OC Coho salmon from 1994 to 2015 (see *Section 1*.*a* in *S1 Text*). We used a D_0_-optimal experimental design [13] to determine attribute levels accounting for multiple correlations and restrictions among the attributes (**Fig 2**; see *Section 1.d in S1 Text* and *Table A in S1 Text* for the permissible combinations of attribute levels). Each survey included three choice experiment questions to increase statistical efficiency in estimation (see *Figure A in S1 Text* for an example). The same status quo alternative is included in every choice question with a $0 cost, along with two conservation scenarios with non-zero costs selected from **Fig 2** –see *Section 2 in S1 Text* for more on value elicitation. For each of the three choice experiment questions, respondents selected one preferred choice, giving us three choice responses per survey respondent. Following standard practice, we assumed respondents answered on behalf of their household.

We used a Tailored Design Method-type approach with four mailings [14] and sent our survey by mail to 5,000 randomly selected residents of the US Pacific Northwest (Oregon, Washington, Idaho, and Northern California {Bay Area and north}) in Fall 2017. This region was targeted because all states have existing runs of Pacific salmon and thus conservation is likely to be salient to these residents. After adjusting for deceased respondents and undeliverable surveys, our response rate was 21% (*Section 1.f in S1 Text*). Oregon State University Institutional Review Board reviewed the survey and research protocol in 2017 and determined the research to be exempt. Consent information was presented in the first mailing and consent is indicated by the respondent’s completion of the survey. Additional information on the survey and the experimental design is provided in the *S1 Text*.

The choice data are analyzed with a random parameters logit (RPL) econometric model that accommodates heterogeneous preferences ([15], *Section 3 in S1 Text*). In particular, we use the following specification to model respondent *i*'s random utility *V* from choosing conservation alternative *k* from choice question *t*:

$$V_{ikt}=-{exp(\beta }_{i1}){Price}_{ikt}+\beta_{i2}{Recovered}_{ikt}+\beta_{i3}{ReturningFish}_{ikt}+\beta_{i4}{Quick}_{ikt}+\beta_{i5}{Fishing1}_{ikt}+\beta_{i6}{Fishing2}_{ikt}+\beta_{i7}{{Recovered}_{ikt}ReturningFish}_{ikt}+\beta_{i8}{{Quick}_{ikt}ReturningFish}_{ikt}+\varepsilon_{ikt},$$ (1)

where *Price*_*ikt*_ is the annual price of the conservation program (in $100s), *Recovered*_*ikt*_ is a binary indicator of whether OC Coho salmon are officially recovered off the ESA (1) or not (0), *ReturningFish*_*ikt*_ represents the increase in the number of returning fish above the status quo baseline of returning OC Coho salmon (in 100,000s of fish), *Quick*_*ikt*_ is a binary indicator of whether returning fish numbers rise quickly (1) or slowly (0), *Fishing*1_*ikt*_ is a binary indicator of whether the OC Coho salmon fishing season is annual with a 5 fish/year limit (1) or not (0), and *Fishing*2_*ikt*_ is a binary indicator of whether the OC Coho salmon fishing season is annual with a 10 fish/year limit (1) or not (0). The status quo is modeled with an alternative-specific constant, *β*_*i*0_ representing the utility of the current state. This random utility modeling framework assumes that each respondent *i* makes the choice *k* that maximizes their utility *V* given the three choices presented to them on choice question *t*.

The RPL model estimates include the mean and standard deviation of the assumed parameter distributions. All random parameters are assumed to be normally distributed. We follow Carson and Czajkowski [16] and exponentiate the price parameter *β*_*i*1_, thereby assuming that exp(*β*_*i*1_) is log-normally distributed. The assumed log-normal distribution of exp(*β*_*i*1_) fixes it to be positive, which–when multiplied by (-1)–precludes respondents from gaining utility from price increases (all else equal). By indexing each parameter by *i* and not *t*, we account for the panel structure of the data by correlating preferences within each respondent and across each choice question that they answer. We use maximum simulated likelihood to estimate the model’s parameters ***β*** with original Matlab code. All parameters in ***β*** are assumed to be random parameters. We construct measures of the central tendency of willingness-to-pay (WTP) for conservation scenarios as an individual’s compensating variation, which is a conventional metric used in benefit-cost analysis (*Section 3.c in S1 Text*).

Stated preference analyses have been criticized for being based on hypothetical choices. Following current best practices [8], we use three approaches in the survey design to minimize hypothetical bias and enhance validity by emphasizing the potential consequentiality of survey response. First, we follow past stated preference literature and use so-called "cheap talk" [17], which reminds respondents to consider their own budget constraints when answering hypothetical questions. In numerous applications, cheap talk has been shown to reduce hypothetical bias (e.g., [18–20]). Second, we frame survey responses as a consequential choice. Respondents are told that the survey is funded by the lead government agency charged with recovering OC Coho salmon (NOAA) and that results will be used to help understand what alternatives the public prefers. Third, we use a binding rather than a voluntary payment vehicle (*Section 2 in S1 Text*).

## Results

### Mean Household Willingness-to-Pay (WTP) estimates for sample

**Fig 3:**
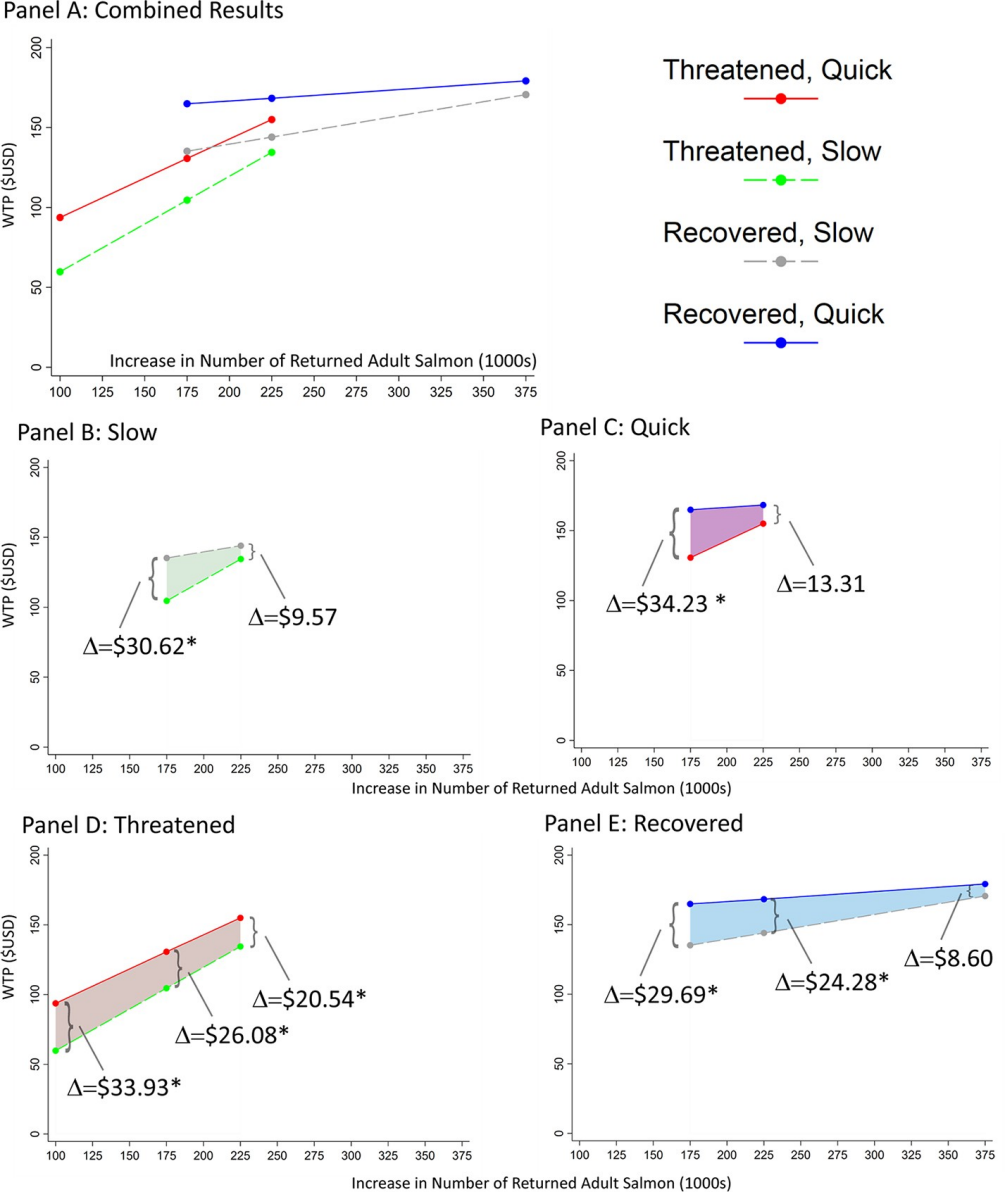
Estimated annual household willingness-to-pay (WTP) functions for Oregon Coast Coho salmon recovery scenarios. The y-axis in each graph depicts average annual household WTP in $USD. The x-axis in each graph represents the increase in numbers of returned adult salmon (in 50-years) from the baseline (in 1000s). A) ESA status and rate of fish return increase held fixed along each line; B) slow rate of fish return increase held fixed, Δ represents marginal change in WTP for “Recovered” ESA status; C): quick rate of fish return increase held fixed, Δ represents WTP for “Recovered” ESA status; D) ESA status of “Threatened” held fixed, Δ represents WTP for “Quick” rate of fish return increase; E) ESA status of “Recovered” held fixed, Δ represents WTP for “Quick” rate of fish return increase. * depicts p<0.05.

Our data include 926 completed surveys consisting of 2,734 usable responses to choice questions. Survey responses to a series of qualitative questions suggest that respondents have heterogeneous preferences regarding changes in the primary survey attributes in **Fig 2** (*Section 3.a in S1 Text*), which supports our choice of the random parameters logit estimator that allows for random respondent heterogeneity. Our estimated parameters from Eq (1) are presented in **Table 1**. Using a likelihood ratio test, the RPL estimation results in **Table 1** are strongly supported over a comparable conditional logit model with no random parameters (log-likelihood = -2576.9) at the 1% level. Results in **Table 1** show that the estimated means of the parameters on *Recovered*, *ReturningFish*, and *Quick* are positive and significantly different from zero (1% level). The estimated standard deviations of most parameters are significantly different from zero (1% level), and thus provide evidence of significant preference heterogeneity across respondents. Results suggest that respondents gain positive utility from de-listing OC Coho salmon from the ESA, from increasing the number of returned salmon, and from doing so quickly (**Fig 3A**; *Section 3.b in S1 Text*). Using a likelihood ratio test, we find no evidence that respondents gain positive utility (on average) from changes in fishing regulations at the 5% level (*Section 3.b in S1 Text*).

**Table 1:** Random parameters logit estimation results for preferred model.

		Parameter	Standard error
Status quo	Alt. Spec. Constant	-11.81*	1.68
Estimated parameter means of conservation alternatives	log(Price)	0.85*	0.14
	Recovered	2.58*	0.61
	ReturningFish	1.70*	0.31
	Quick	1.18*	0.37
	Fishing1	-0.25	0.16
	Fishing2	-0.34	0.27
	Recovered*ReturningFish	-0.88*	0.26
	Quick*ReturningFish	-0.22	0.17
Estimated parameter standard deviations of conservation alternatives	St. Dev.(Price)	1.66*	0.07
	St. Dev.(Recovered)	1.17	1.00
	St. Dev. (ReturningFish)	0.88*	0.21
	St. Dev. (Quick)	0.78*	0.32
	St. Dev. (Fishing1)	1.04*	0.44
	St. Dev. (Fishing2)	0.28	0.93
	St. Dev. (Recovered*ReturningFish)	0.61*	0.21
	St. Dev. (Quick*ReturningFish)	0.53*	0.19
	St. Dev. (ASC)	10.81*	1.42
	Log-likelihood	-2016.16	
	N	2,734	

* indicates statistically significant at the 1% level

Price is measured in $100s; ReturningFish is measured in 100,000s of returning adult fish.

Respondents had mean annual willingness-to-pay (WTP) for attributes of OC Coho salmon recovery that vary depending on the recovery scenarios (**Fig 3A**, *Table B in S1 Text*). The lowest mean household WTP is approximately $60/y for the least aggressive scenario of 100,000 more returning fish, with a slow increase, and remaining at an ESA status of “threatened.” The highest mean WTP is $179/y for the most aggressive scenario of 375,000 more returning fish with a quick increase that achieves an ESA status of “recovered.”

We use estimates of WTP to calculate the marginal values for individual attributes, holding other attributes fixed. People were willing to pay more for most attributes that indicated improvement in the OC Coho salmon stock, conditional on the magnitude of other attributes, and most of these marginal changes were significantly different from zero (**Fig 3B–3E**; *Table C in S1 Text*). For example, people were WTP an average of $31/y more for the species to be recovered off the ESA, dependent on 175,000 more fish returned (**Fig 3B**). If the returns increase to 225,000 more fish, WTP for recovery decreased to an average of $10/y per household; WTP increased (by $34/y and $13/y, respectively) when recovery was characterized as quick (**Fig 3C**). When the species remains threatened under the ESA with a modest return of 100,000 more fish, people were WTP an average of $34/y more for “quick” versus “slow” increases, but that amount decreased with greater fish returns (**Fig 3D**). Finally, we also found WTP estimates per unit of extra returning fish were notably lower when OC Coho salmon are recovered off the ESA (slope of the line in **Fig 3E**). Values for a quick rather than slow rate of return diminished with the number of fish returned ($30/y for 175,000 fish vs. $9/y for 375,000 fish).

The slope of the linear lines in **Fig 3** depict the WTP for an extra 100,000 fish, conditional on the magnitude of the other attributes. If the species remains threatened, then the WTP per 100,000 returning fish is $59.75/y if the rate of change in returning fish is slow–see *section 1*.*a* in *S1 Text* for a precise mathematical definition of rate of change. If the rate of change in returning fish is quick, then the WTP per 100,000 returning fish falls to approximately $49.28/y. If the species is recovered, the WTP per 100,000 returning fish falls to approximately $11.77/y for a slow rate of change and $4.55 for a quick change (*Table C* in *S1 Text*). While the points in **Fig 3** represent scenarios from our experimental design (and are presented in *Tables B and C* in *S1 Text*), other conservation scenarios outside of our experimental design could be valued using these marginal attribute values. For example, the linear WTP function from **Fig 3** for the scenario where the rate of increase is slow and the species is threatened can be expressed as *WTP*|*Slow*,*Threatened* = $59.75*ReturningFish, where *ReturningFish* is measured in 100,000s of fish. For the scenario where the rate of increase is quick and the species is threatened, the linear WTP function has a different y-intercept and slope: *WTP*|*Quick*,*Threatened* = $44.41+$49.28*ReturningFish. Therefore, the average household WTP for a small conservation investment that generates 10,000 more returning fish above the baseline would be $5.98/y if the rate of increase were slow and $49.34/y if the rate of increase were quick.

We subjected our preferred model to a series of validity checks consistent with best practices for stated preference research [8] and found that our results are robust to potential protest respondents, attribute non-attendance, and the stratified sampling scheme (*Sections 3.d-3.f in S1 Text, Tables D–K in S1 Text*).

### Aggregation of Willingness-to-Pay (WTP) to represent population benefits for Pacific Northwest residents

Our WTP results can be used to represent population-level non-market benefits of conservation efforts aimed at increasing the amount of returning OC Coho salmon. Appropriate aggregation requires multiplying a population-level mean WTP by the overall respective population size. To make these estimates, we analyzed the representativeness of our data in order to determine the generalizability of our sample WTP estimates to the Pacific Northwest population as a whole.

First, using supplemental and comparative demographic information about all Pacific Northwest residents and our survey respondents, we assess observable differences between the sample and the broader population of Pacific Northwest households (**Table 2**). The broader population data comes from the U.S. Census Bureau (2017). While our survey respondents were representative of the broader population in terms of median household income, they were more likely to be older, white, and educated men than the broader population of the region. As such, we re-estimated variants of our primary model in Eq (1) which included interactions between the demographic variables in **Table 2** and the key utility parameters representing preferences for OC Coho salmon conservation: the recovery status of OC Coho salmon (*β*_*i*2_), the number of returning adult salmon (*β*_*i*3_), and whether their returns were quick (*β*_*i*4_). We then conducted a series of likelihood ratio tests for the null hypothesis that there is no significant interaction between utility parameters and the key demographic variables, with corresponding p-values presented in the final column in **Table 2**. Results show significant utility preference differences between those with a 4-year college degree compared to those without a degree (1% level). In particular, college graduates are WTP approximately $31/y more per 100,000 returning fish than those without a college degree. The final column in **Table 2** shows p-values well above 0.05 for likelihood ratio tests involving the other demographic characteristics, which indicates no evidence of significant preference differences across older versus younger adults, and across gender or race. Therefore, aggregation of our WTP results to the broader population requires adjusting our preference parameters to represent the population share of college graduates–see *Section 3*.*g* in *S1 Text* for details.

**Table 2:** Sample demographic characteristics versus population characteristics of Pacific Northwest (PNW) residents for the Oregon Coast Coho salmon stated preference survey. P-values are for the null hypothesis that demographics do not interact with utility parameters.

Demographic Statistic	Sample	Pacific Northwest Population	Is Sample Representative?	p-value for utility interaction
Household Median Income	$60k - $80k	$70k	Yes	-
Percent College Grads	54.0%	35.8%	No	0.00
Percent Age 65+	38.5%	15.1%	No	0.92
Percent Male	61.1%	49.8%	No	0.87
Percent White	87.7%	72.0%	No	0.29

All demographic statistics for the Pacific Northwest population taken from the U.S. Census Bureau (2017); P-values for the null hypothesis that demographics do not interact with utility parameters are estimated with a likelihood ratio test.

Second, we analyze potential sample selection bias in terms of unobservable preferences for salmon conservation. Given there are no formal sample selection corrections for non-linear logit models like ours, we follow the approach of Cameron and DeShazo [21] and Kolstoe and Cameron [22], which is recommended as part of current best practices [8]. The approach first estimates a binary choice econometric model of the propensity of individuals to respond to our survey (a selection equation) based on observable variables available for both respondents and non-respondents–demographic data for the census tracts or county where each individual lives, state dummy variables, and information about the mail delivery system for each individual. We find evidence that households with neighborhood delivery and collection box units have a significantly lower propensity to respond to our survey (10% level), possibly because the outgoing mail slots in such units may be too small for returning our large survey packets. Therefore, the mail delivery variables proxy for household costs of returning our large survey packet and thus serve as an exclusion restriction for our selection equation. We then generate a variable (*pdiff*) that represents the difference between the estimated propensity for each individual to respond to the survey and the average propensity to respond. Finally, we interact *pdiff* with the key utility parameters representing preferences for OC Coho salmon conservation (*β*_*i*2_, *β*_*i*3_, *β*_*i*4_) to test whether individuals with a higher estimated propensity to respond to the survey have systematically different preferences for OC Coho salmon conservation. A likelihood ratio test fails to reject the null hypothesis that preferences do not vary with *pdiff* (*p* = 0.56)–full results are presented in *Section 3.g in S1 Text* (*Tables M and N in S1 Text*). We therefore find little evidence of sample selection bias based on unobservable preferences.

Given the two analyses of sample selection bias based on observable and unobservable factors, we present results from two approaches to value aggregation to indicate sensitivity to aggregation assumptions. First, we use a lower-bound approach [23] for value aggregation and use the survey response rate as the portion of the population for which WTP is non-zero (*Section 3.g in S1 Text*). We use this approach to generate an estimate of aggregate value, differentiating the response rate by Oregon (25.4%) and non-Oregon (17.8%) households. Our sample WTP estimates are then multiplied by 398,889 Oregon households (0.254×1,570,430 households) plus 1,395,098 non-Oregon Pacific Northwest households (0.178×7,837,629 households). This conservative approach is useful in the case when the respondents answered the survey based on some unobservable preferences for salmon conservation (although we provide evidence that this is unlikely in our analysis). Second, our upper-bound approach builds off our evidence regarding sample selection bias and uses the population-adjusted mean WTP estimate multiplied by the total population (9,408,059 households) to obtain the population level benefits (see S*ection 3.g in S1 Text*). **Table 3** shows the results from the two approaches for value aggregation and indicates large aggregate benefit estimates–see also *Figures E and F in S1 Text*. The lower bound (upper bound) approach to aggregation reveals annual benefit estimates of $107 million ($518 million) for the most pessimistic conservation scenario and $321 million ($1.46 billion) for the most optimistic scenario. We also calculate population benefit estimates for changes in individual attributes of OC Coho salmon conservation using both the lower bound and upper bound approaches to value aggregation (*Table P* in *S1 Text*). We find that benefits to the population of Pacific Northwest residents are as much as $518 million/y for an extra 100,000 returning salmon and as much as $277 million/y for quick vs. slow increases in returning fish.

**Table 3:** Estimated annual mean household Willingness-to-Pay (WTP) and aggregate population benefits for Oregon Coast Coho Salmon conservation scenarios.

OC Coho Salmon Conservation Scenario			Household WTP ($)		Population Benefits ($)	
ESA status	Change in number of returning OC salmon (fish)	Rate of change in number of returning OC salmon	Sample mean WTP ($)	Population-adjusted mean WTP ($)	Lower bound benefits = sample mean WTP x 1,793,987 households	Upper bound benefits = population-adjusted mean WTP x 9,408,059 households
Threatened	100,000	Slow	$59.75	$55.13	$107 million	$518 million
	175,000		$104.57	$96.47	$188 million	$908 million
	225,000		$134.45	$124.04	$241 million	$1.17 billion
	100,000	Quick	$93.69	$84.62	$168 million	$796 million
	175,000		$130.65	$118.29	$234 million	$1.11 billion
	225,000		$154.99	$140.43	$278 million	$1.32 billion
Recovered	175,000	Slow	$135.19	$123.45	$243 million	$1.16 billion
	225,000		$144.02	$130.04	$258 million	$1.22 billion
	375,000		$170.58	$150.08	$306 million	$1.41 billion
	175,000	Quick	$164.88	$149.29	$296 million	$1.40 billion
	225,000		$168.29	$150.53	$302 million	$1.42 billion
	375,000		$179.19	$155.30	$321 million	$1.46 billion

95% confidence intervals for Sample mean WTP are found in *Table B* in *S1 Text*; 95% confidence intervals for Population-adjusted mean WTP are found in *Table O* in *S1 Text*.

## Discussion

This study shows that non-market, non-consumptive values provided by investments in natural capital can be both incremental in nature and dynamic through time. By quantifying the marginal change in non-consumptive value for a threatened species of salmon, we find that public preferences extend beyond just meeting a non-marginal recovery threshold. Our results suggest that the public places significant economic value not just for official recovery status, but also for higher return numbers of salmon, and for increases that are quick rather than slow.

We find that the average household WTP for the most ambitious recovery program–one that involves OC Coho salmon reaching recovered status under the ESA–is $179/y. Upon aggregating to the broader population of PNW residents, the WTP for this most ambitious recovery program ranges from a lower bound of $321 million/y to an upper bound of approximately $1.46 billion/y depending on aggregation assumptions. Given that the most ambitious recovery program in our experimental design is based on the OC Coho Conservation Plan for the State of Oregon [10], the population benefit estimates represent the non-market economic value associated with successfully implementing this state-level conservation plan. Importantly, we also find that the public has significant WTP for habitat restoration programs that generate much smaller changes in salmon abundance, even for programs that do not result in the stock becoming de-listed from the ESA. For example, the average household WTP of approximately $60/y for the least ambitious scenario in our experimental design (100,000 more returning fish with no change in the threatened status under the ESA) still produces between $107 million/y (lower bound) to $518 million/y (upper bound) in non-market economic benefits (**Table 3**). Given that no ESA-listed species of Pacific salmon have been de-listed as of 2018, our results provide evidence that the public values ESA conservation activities that have yet to achieve a recovered status for their target species. Measuring the benefits of incremental improvements to ESA-listed species may take on added relevance given current priorities of the U.S. Department of Interior, which announced in summer 2018 that it intends to abandon the longstanding policy of ignoring economic impacts when making listing and de-listing determinations [24]. Further, by finding sizable public benefits of incremental improvements, our results provide support for the assertion that debates about ESA-effectiveness should be based on criteria such as partial gains in threatened species management, rather than strictly on whether species have been, or should be, officially de-listed ([1], [25]).

A second important and novel finding from this study is clear quantitative evidence that the public prefers receiving non-market benefits from OC Coho salmon sooner rather than later. Holding other conservation attributes constant, the average household WTP for a quick rate of increase in salmon returns is up to $34/y (**Fig 3**), contingent on the levels of the other program attributes. Depending on aggregation assumptions, the highest estimated population benefits associated with a quick rate of increase in salmon ranges between $60 million/y (lower bound) and $277 million/y (upper bound) (*Table P in S1 Text*). The practical relevance of the result is that there are significant economic benefits to the public from natural capital investments that deliver on their objectives quickly, rather than gradually. The WTP for a quick increase in returning salmon is diminishing in the number of returning fish, and is lower for scenarios that generate a recovered rather than a threatened ESA status (**Fig 3**; *Table P in S1 Text*).

In general, the value provided by protecting threatened and endangered species is non-market in nature [2]. As Polasky et al. [26] point out, current research that either maps trade-offs among natural capital and other forms of wealth, or quantifies trends in implicit natural capital asset prices, tends to lean heavily on linked market activity such as commodity or land market transactions. The disconnect between these methods and non-market valuation is noted by their developers, who cite the need for non-market valuation research and improved integration with quantitative measures of natural capital ([27] p. 8; [26] p. 455). Pricing natural capital ensures that it is not undercounted or ignored outright in regional or national economic assessments [28]. To help prioritize new investments such as habitat restoration, there is also interest in applying economic benefit-cost methods to natural capital ([27–30]). In order to include threatened species recovery in either exercise–pricing or benefit-cost analysis–a monetary estimate of the public benefit generated by helping threatened and endangered species over time is essential. Our approach offers a way to measure the incremental and dynamic non-market public benefit yielded by investment in natural capital that is generally unavailable to conservation practitioners ([7], [31–33]).

Quantitative survey methods remain the leading methodology for measuring non-market values provided by protecting threatened and endangered species. While our analysis focuses on OC Coho salmon, the experimental design could be adapted to uncover more insights into public preferences for other threatened species, along with other types of natural capital. The challenge of incorporating non-market values is perhaps even greater for research focused on the optimal management of natural capital and related forward-looking exercises that involve predicting outcomes that differ substantially from the status quo ([34–36]). These studies need to explore a wide range of possible outcomes and use that information to predict as accurately as possible the associated flows of non-market values in order to identify realistic and cost-effective natural capital allocations over time and across space ([37–39]). Our stated preference approach provides an integral step forward in quantifying the multiple dimensions of public benefits that arise from natural capital investments.

## Supporting information

S1 Text The supporting information is organized to show how the study design meets all contemporary best practices for conducting stated preference studies, including survey development and implementation, value elicitation, and data analysis. (DOCX)
