# Supplementary material for: The non-market benefits of early and partial gains in managing threatened salmon
Source: PLoS One. 2019 Aug 14;14(8):e0220260. doi: 10.1371/journal.pone.0220260 (PMC6693736; doi:10.1371/journal.pone.0220260)
Supplement: S1 Text — (DOCX) [file pone.0220260.s001.docx]

Supporting Information Text (S1 Text) for

**The non-market benefits of early and partial gains in managing threatened salmon**

David J. Lewis^1*^, Steven J. Dundas^1, 2^, David M. Kling^1^, Daniel K. Lew^3^, Sally D. Hacker^4^

1. Department of Applied Economics, Oregon State University, Corvallis, Oregon, United States of America.
2. Coastal Oregon Marine Experiment Station, Oregon State University, Newport, Oregon, United States of America.
3. Alaska Fisheries Science Center, National Oceanic and Atmospheric Administration, Seattle, Washington, United States of America.
4. Department of Integrative Biology, Oregon State University, Corvallis, Oregon, United States of America.

*Corresponding Author: David J. Lewis, [lewisda@oregonstate.edu](mailto:lewisda@oregonstate.edu)

**This file includes (in order):**

Supplementary text

Figs. A to F

Tables A to P

References for S1 reference citations

Supporting Information

The supporting information is organized to show how our study design meets all recommendations and contemporary best practices from Johnston et al.’s (2017) paper on conducting stated preference studies. We address how our choice experiment research design follows each recommendation as a means of establishing the study's content validity.

**Section 1. Survey development and implementation**

*1.a Scenario descriptions*

The foundation for measuring non-market values for Oregon Coast (OC) Coho salmon recovery from stated preference surveys is a set of realistic habitat restoration scenarios, including a baseline against which changes are measured. Calculations for returning adult OC Coho salmon are made using the Oregon Department of Fish and Wildlife’s (ODFW) Salmon Recovery Tracker website (<http://www.odfwrecoverytracker.org/>), which provides annual estimates for returning adult OC Coho salmon from 1994 to 2015. We use two primary documents to guide our scenario development: the State of Oregon’s OC Coho Conservation Plan and the Federal Government’s Recovery Plan for OC Coho which was developed by the National Oceanic and Atmospheric Administration (NOAA).

The baseline number of returning adult OC Coho salmon is presented to survey respondents as a flat line fixed at 150,000 fish. This number was calculated using the 22-year average of returning OC Coho salmon from 1994 to 2015, which was 152,187 fish. We justify the constant baseline of returning fish based on NOAA’s Oregon Coast Coho Recovery Plan, which was released in December 2016 and continues to support the statement from the 2011 listing decision that finds “little evidence for an overall improving trend in freshwater habitat conditions since the mid-1990s” (p.4-10, 4-11).

Habitat restoration scenarios that increase the number of returning OC Coho salmon are given to survey respondents in the choice experiment. The first attribute of each scenario is Endangered Species Act (ESA) status – threatened or recovered. The second attribute is number of returning adult fish – 150,000; 250,000; 325,000; 375,000; or 525,000 fish. The size of 150,000 returning fish is the baseline, and is presented in every choice question as the status quo number with minimal additional conservation activities. The largest size is 525,000 fish, and is calculated by using the conservation goals set in the State of Oregon’s Oregon Coast Coho Conservation Plan of 2007, hereafter the State’s Plan. The State’s Plan presents goals of returning fish that are dependent on ocean conditions. The goals are 101,000 fish (extremely low ocean conditions), 371,000 fish (low conditions), 715,000 fish (medium conditions), and 817,000 fish (high conditions). We create a weighted average of the returning fish goal using the observed distribution of ocean conditions from 1994 to 2015, which we calculate as 517,455 returning fish. We round to 525,000 fish to get our most optimistic recovery scenario to present to respondents. Given the baseline and most optimistic scenarios, we define three middle ground returning fish numbers to develop experimental variation: 250,000, 325,000, and 375,000 fish.

An important feature of the scenarios we present to respondents is the rate with which returning salmon increase from the baseline number to the final number of returning fish for each scenario. We show each survey respondent a graph depicting the rate of increase in the number or returning fish – see Figure 2 in the main text for an example – which are generated using the beta function (Yin et al. 2003, p.368, equation 11):

$RetFish\left( t \right)=RetFish(0)+\left( RetFish(T)-RetFish(0) \right)\left( 1+\left[ \frac{T-t}{T-\tau} \right] \right)\left( \frac{t}{T} \right)^{\frac{T}{T-\tau}}$ (S1)

Here $RetFish\left( t \right)$ is the number of returning adult OC Coho salmon in time *t*, the parameter $T$ is the terminal time, the initial time is *t*=0, and $\tau$ is a parameter that tunes the timing of the maximum rate of change. The initial number of returning fish $RetFish\left( 0 \right)$ is always set at the baseline of 150,000 returning fish, while $RetFish\left( T \right)$ is set at the final number of returning fish in $T=50$ years (250,000; 325,000; 375,000; or 525,000). The beta function creates an S-shaped (sigmoidal) time path of increase in the number of adult OC Coho salmon, and alternative values of $\tau$ can be used to change the rate with which the number of returning fish changes. For any given fish abundance in the terminal time, we use the value of $\tau=38.7$ to depict a slow path, and $\tau=13.7$ to depict a quick path. Figure 2 in the main text shows an example of a slow path and a quick path to $RetFish(T)$=525,000 fish.

*1.b Survey pre-testing*

We conducted two formal focus groups in Sacramento, California, and Portland, Oregon. Both formal focus groups were conducted at professional survey research centers, and recruits were selected to broadly represent the general population of residents. Both focus groups contained one moderator and two observers. In addition, we conducted two informal focus groups by showing the survey to a graduate class at Oregon State University and to an external advisory board convened for a funded NOAA project. Further, various drafts of the survey were shown to numerous other individuals, including salmon biologists, the lead author of the NOAA recovery plan (Rob Walton), survey design specialists, and other members of the general public. Blaine Bellerud from NOAA was kind enough to share some illustrations of Coho salmon and their habitat that we ended up using in the survey. All focus groups and informal conversations led to a variety of changes in the layout and structure of the survey, especially with regards to the clarity of the early information presented in the survey. An important suggestion from focus group testing was to omit stating numerical goals for returning OC Coho salmon in information before the choice questions are presented, as numerous focus group participants appeared to be anchoring off numerical goals stated before the choice questions. The final version of the survey does not state numerical goals of returning fish until the choice questions.

A limited pilot version of the survey was conducted in the summer of 2017, which involved administering the survey to 150 randomly selected respondents from the study region. Our goals for the pilot were i) examine respondents’ ability to understand the survey, ii) evaluate the performance of the survey protocols and approximate the expected response rate, iii) evaluate whether the highest cost presented to respondents was sufficient to “choke off” demand, and iv) modify the survey if problems were found. The pilot was conducted with an address-based sample of households obtained from the US Postal Service through the Genesys Sampling Company. Only one copy of the survey was sent to each respondent, along with a pre-notification letter and a post-card reminder. The pilot study had three versions of the choice questions in a highly simplified experimental design. Response rate for the pilot was only 11.7%, which prompted a shift in sampling away from an “address-based” sample without individual respondent names, to a final survey sampling approach in which all mailings included the first and last names of respondents to aid in personalizing the surveys, which can improve response rates (Dillman et al. 2007). The response rate for the full survey was much improved at 21.5%. The pilot survey otherwise provided confidence that the survey was understandable and that $350 was sufficiently large for the highest cost attribute level.

*1.c Attribute-based choice experiment approach*

We selected the choice experiment method, an attribute-based value elicitation approach, for use in designing the survey (Louviere et al. 2000). The choice experiment approach was motivated by a desire to value marginal changes in returning adult OC Coho salmon numbers. The list of attributes and their levels is presented in Figure 2 of the main text. The levels of the ESA Status and number of returning fish attributes are discussed above. Since only improved conditions were considered in each choice question, the levels of all attributes in the habitat restoration scenarios are at least as good as the attributes in the status quo. The “Number of returning fish over time” attribute was included to examine willingness-to-pay for conservation activities that occur up-front in early years versus a more gradual approach. The “Fishing regulations” attribute was included to examine willingness-to-pay for more recreational fishing opportunities from recovering fish, where the most lax regulation is pegged to current regulations in place for Chinook salmon on the Oregon Coast. The “Cost to respondents” attribute is the key attribute used to translate preferences to a money metric.

*1.d Experimental design*

Our approach to experimental design uses a D_0_-optimal design (Huber and Zwerina 1996) with multiple restrictions and imposed correlations across attribute levels (e.g., Johnson et al. 2013). In particular, ESA status, number of returning fish, and fishing regulations are necessarily correlated in any realistic conservation scenario. Higher fish returns are more likely to be moved from threatened to recovered ESA status. Fishing regulations are more likely to be relaxed with higher fish returns and a recovered ESA status. Our experimental design ensures that no choice could dominate or be dominated within or across choice questions within the same survey. Also, no single survey is allowed to include a single returning fish level that is coincident with both a threatened and recovered ESA status within the same survey.

The experimental design analysis begins by considering the full factorial of attribute levels within a choice question, consisting of 2×5×3×3×9 = 810 possible combinations of attribute levels. Many of these combinations are not reasonable for this application due to the necessary correlations discussed above, and the rules presented in Table A governed the permissible combinations of attribute levels.

The status quo is included in every choice question, and includes the following codes: ESA Status (1), Number of returning fish (1), Number of returning fish over time (Rate) (1), Fishing regulations (1), and Cost (1). All scenarios are allowed to include any of the eight non-zero cost options, and restricted to the combinations set in Table A. Any choice question that violates the allowable combinations in Table A is discarded, leaving 177 unique combinations of attributes for the scenarios (not including the status quo).

Every choice question has a status quo and two conservation alternatives (A and B). There are 31,152 (177×177 – 177) unique combinations of two distinct choice alternatives from which to choose. Many of these combinations include choices that are dominated – e.g., all levels of choice A are greater than or equal to all levels of choice B and the cost of choice A is less than the cost of choice B. Discarding all dominant choices leaves 15,392 unique choice questions from which to select the experimental design. We create a 15,392 × 15,392 matrix indicating whether each combination of choice questions is incompatible (1) or compatible (0). Incompatible choice questions have at least one dominated choice across choice questions, or have different ESA status levels (threatened or recovered) across the same size of returning fish, or have a threatened status level for a fish return number that is larger than the recovered fish return number in the other choice question.

Our survey design includes 20 survey versions, with 3 choice questions per version. Given the set of acceptable choice questions, we form a complete survey design by drawing 20 random combinations of three unique choice questions each that satisfy all of the above rules, and then calculate the D_0_-error from a random utility specification in a multinomial logit model with main effects and an interaction between both ESA status and fish return number, and between fish returns and rate of increase in fish returns. The status quo is represented with an alternative specific constant. We repeat the D_0_-error calculation for 100,000 randomly created survey designs that satisfy the above rules, and rank each possible survey design in ascending order by the D_0_-error score.

For each of the top 20 experimental designs from above (lowest D_0_-error), we conduct a Monte Carlo simulation analysis with varying true values of the random utility parameters to examine parameter estimates and significance of parameter estimates. For each design, we assume a set of true parameter values – and hence, willingness-to-pay for marginal changes in each attribute – and conduct 1,000 separate estimations. We average over the 1,000 separate estimations to examine estimator consistency, and then examine the percentage of simulated estimations in which the parameters are statistically significant from zero (5% level). This approach relaxes the assumption that parameters are zero in the D_0_-optimal design. Our main criteria to find the best design (from among the top 20 most D_0_-efficient designs) was to find the design that had the highest percentage of simulations where all parameters were identified as statistically significant at the 5% level. The top D_0_-optimal design was also deemed the best design in the Monte Carlo analysis. For the most plausible set of true parameter values, the best design was able to generate parameter estimates which were significantly different from zero (5% level) on at least 99% of simulations for all parameters.

*1.e Data collection and human subjects protection*

Our survey design was examined by Oregon State University Institutional Review Board in 2017, and received a notice of exemption (OSU Proposal #NA16OAR4320152). There was no deception used in the survey design.

|  |
| --- |

*1.f Sampling*

We sent our survey to 5,000 randomly selected respondents chosen from the general population of residents in Oregon, Washington, Idaho, and Northern California (Bay Area and north). Of the 5,000 surveys sent, 2,500 were sent to Oregon residents and 2,500 were sent to residents of the other four states. We chose a mail survey so that the presentation of choice questions could be standardized and not subject to different internet browser sizes and formatting. The survey was administered following a Tailored Design Method-type approach with four mailings (Dillman et al. 2014). We sent a notification letter to named respondents on 9/14/17 which described the survey, the importance of salmon conservation as a public policy issue, and named the National Oceanic and Atmospheric Administration (NOAA) as both the funder of our survey, and as the main agency charged with recovering OC Coho salmon under the ESA. The full 16-page survey was mailed on 9/20/17, with a reminder/thank you mailed on 9/29/17. For those who didn’t respond to the initial survey mailing, a second survey was mailed on 10/19/17. All completed surveys returned by 12/14/17 were included in the final dataset used for estimation. Our response rate was 21.5%, using the RR4 method developed by the American Association for Public Opinion Research (AAPOR 2016). Our application of the RR4 method deemed the following respondents as ineligible: deceased (n=38), otherwise unable to respond (n=5), those surveys returned by the US Post Office as ‘undeliverable’ (n=581). Using the RR1 method (AAPOR 2016) which includes all undeliverable surveys in the denominator of the response rate calculation, the response rate is 19%. Since undeliverable surveys were never received by a respondent, we view them as never having the opportunity to participate and thus find the RR4 method appropriate.

**Section 2. Value elicitation**

Each survey contains three choice experiment questions in order to increase overall statistical efficiency across the sample in estimation. Given that our survey is 16-pages long, we were concerned that more than three questions would make the survey too long and hurt response rates. The three question format was chosen with random ordering of the questions within each survey and checks that no single choice is dominated or dominant across and within each choice question. The same status quo alternative is included in every choice question with a $0 cost, along with two conservation scenarios with non-zero costs. Following advice from Johnston et al. (2017) with regards to choice experiments, we do not include a no-answer option in each choice question. Figure A presents an example choice question from the final survey design. The payment vehicle used in our survey is two-part. We tell respondents that federal and/or state taxes will increase to fund the OC Coho salmon habitat restoration program, and that lumber and/or agricultural prices may increase as a result of restrictions placed on land management activities. A similar combined tax and price payment vehicle is used in Lew et al. (2010), Lew and Wallmo (2011), MacDonald et al. (2011), and Wakamatsu et al. (2018).

The survey includes a number of auxiliary and supporting questions. For example, we ask questions to probe about the salience of salmon conservation to respondents. We ask whether respondents think the main attributes used in the choice questions are important or not in a manner that helps understand possible non-attendance that respondents may have by ignoring certain attributes. We also ask questions that probe whether respondents may protest the mechanism used in the survey. We also ask questions that aim to quantify the underlying discount rate that respondents may use to compare benefits today with benefits in the future, as a way of examining potential heterogeneity respondents may hold for the rate of change in returning fish.

**Section 3. Data Analysis**

*3.a Qualitative evidence of preference heterogeneity*

Selection of an estimation model for analyzing our stated preference data should be influenced by any evidence for preference heterogeneity with our respondents. In the main text, we present qualitative evidence of heterogeneous respondent preferences for numerous survey attributes based on responses to several auxiliary survey questions. Most respondents (>85%) thought that it was either very important or somewhat important to recover OC Coho salmon, to recover the species within 50 years, and to increase the number of returning fish by as much as possible (Figure B a-c). There was less support for relaxing fishing regulations (~ 60%) and 30% of the respondents thought it unimportant to allow recreational fishing every year (Figure B.d). We also found that 75% of respondents somewhat or strongly agreed with the statement that “even if it costs us more money, we should do more so that the Oregon Coast Coho salmon recovers” (Figure B.e). There was more heterogeneity with preferences for a quick vs. slow rate of increasing returning fish, as only 57% of respondents somewhat or strongly agreed with the statement that “even if it costs us more money, we should try for a quick change rather than a slow change in Oregon Coast Coho salmon”, while 40% responded that they “neither agree or disagree” or “somewhat or strongly disagree” (Figure B.f).

An additional aspect of heterogeneity in WTP for quick vs. slow changes in numbers of returning OC Coho salmon is in the discount rate that respondents implicitly use to discount future benefits compared to current benefits. Figure C shows discount rate estimates from a thought experiment in our survey where we asked about respondent’s preferences for receiving a $100 check today vs. a larger check one year from now. We find evidence of heterogeneity in the implicit discount rate that respondents use.

*3.b Choice of econometric estimator – Random parameters (mixed) logit*

We choose to estimate willingness-to-pay by implementing a random parameters logit (RPL) representation of a random utility discrete-choice model (Train 2009). Our choice of the RPL model is based on our strong qualitative evidence of heterogeneous preferences (especially for increasing salmon returns quickly and for relaxing fishing regulations), and because Johnston et al. (2017) argue that contemporary analyses of stated preference data should allow for unobserved preference heterogeneity as a best practice. The primary specification is presented as equation (1) in the main text, and parameters from estimating equation (1) using maximum simulated likelihood are presented in Table 1 in the main text.

Results indicate that we fail to reject the null that the mean parameters for relaxing fishing regulations (*Fishing1*, *Fishing2*) are significantly different from zero (5% level). Further, a likelihood ratio test fails to reject the null that all parameters on the fishing regulations variables (*Fishing1*, *Fishing2*) are jointly zero (5% level). Thus, estimation results indicate that respondents gain positive utility from recovering OC Coho salmon (*Recovered*), increasing number of returning fish (*ReturningFish*), and increasing the number of returning fish quickly (*Quick*). We find little evidence that respondents gain positive utility from relaxing fishing regulations.

*3.c Value estimation for household WTP*

To measure household WTP, we assume that the marginal utility of income is constant, and therefore WTP is measured by an individual’s compensating variation: $CV=\left( 1/{-\gamma} \right)[V^{1}-V^{0}]$, where $V^{0}$ is the conditional indirect utility of a baseline state of the world without OC Coho salmon conservation and $V^{1}$ is the conditional indirect utility of a different state of the world with a particular OC Coho salmon conservation scenario. The parameter $\gamma$ is the marginal utility of income, which is the cost parameter in our random utility model. We use parameter estimates from Table 1 in the main text to calculate estimates of the central tendency of household WTP and confidence intervals. Given the use of random parameters, we simulate household measures of WTP using standard ratios of coefficients for alternative conservation scenarios. Further, we use the Krinsky-Robb method to estimate 95% confidence intervals for our measures of WTP (Krinsky and Robb 1986). Table B presents average household WTP estimates for the allowable combinations of attribute levels from Table A. Table B presents the numerical values behind Figure 3 in the main text.

All estimated household WTP values in Table B are significantly larger than zero at the 5% level. The lowest WTP in the table is $59.75/year for the most modest conservation scenario of 100,000 more returning fish, slow fish return increase, and an ESA status of “threatened”. The highest WTP in the table is $179.19/year for the most aggressive conservation scenario of 375,000 more returning fish with a quick timeline and an ESA status of “recovered”. Fishing regulations are unchanged from the status quo in Table S3 because we fail to reject the null that all estimated utility parameters on fishing regulations are jointly zero in section 3b.

We also use parameter estimates to calculate estimates of the central tendency of household WTP and confidence intervals for marginal changes in individual attributes, holding other attributes fixed (Table C). Table C presents numerical values for values presented in Figure 3 of the main text.

The main results are consistent with economic theory and evidence from past studies which establish that the general public is willing-to-pay to recover endangered species (Richardson and Loomis 2009; Lew 2015). Our results demonstrate sensitivity to scope, as respondents prefer more returning OC Coho salmon, and respondents prefer a quick rate of increase in fish returns compared to a slow rate. Thus, our findings of sensitivity to scope suggest construct validity in our stated preference exercise.

*3.d Analysis of potential behavioral response anomalies – Potential protests*

The effect of behavioral “anomalies” on stated preference analyses has been the subject of significant investigation in recent literature (Carson 2012). As a best practice, Johnston et al. (2017) suggest that analysis of stated preference data should examine the role of potential behavioral response anomalies on estimates of WTP. Two important anomalies that we focus on are the role of so-called protest respondents, and attribute non-attendance. Protest responses arise from a respondent rejecting the valuation scenario in some way, such that the researcher is not confident that their responses reflect their preferences (Meyerhoff and Liebe 2010). Attribute non-attendance arises when respondents ignore certain attributes in making their choices (Scarpa et al. 2009).

We identify potential protest responses through responses to auxiliary questions carefully designed to evaluate the extent to which respondents may be rejecting key parts of our valuation exercise. Figure D presents responses to a set of three auxiliary questions that were asked after respondents answered the choice questions. We identify potential protest respondents as the 177 respondents who answered “strongly disagree” to Q16a or “strongly agree” to either Q16b or Q16d.

We then re-estimated the primary model without the potential protest respondents with the results in Table D, along with the results of the full sample for comparison. Qualitative signs are the same across the two samples, but one notable difference is the reduction in the estimated standard deviation of the parameters in the sample without the potential “protest respondents”.

We also estimate WTP for the OC Coho salmon conservation programs for the full sample and the sample without protest respondents in Table E. We see that WTP estimates are somewhat higher when we drop potential protest respondents. However, using the 95% confidence intervals from the sample without potential protest respondents, we almost always fail to reject the null hypothesis that the mean WTP estimate in the protest sample is equal to the comparable point estimate in the full sample. We conclude that potential protest respondents do not have a large impact on the estimation results, and thus retain all data for estimation.

*3.e Analysis of potential behavioral response anomalies – Attribute Non-Attendance*

A second behavioral response anomaly that we analyze is the potential for attribute non-attendance. Attribute non-attendance arises when respondents ignore certain attributes in making their choices, which is observationally equivalent to respondents holding zero marginal utility for changes in an attribute (Scarpa et al. 2009). Two attributes in particular may yield significant non-attendance in our model. Using the preferred model above, we fail to reject the null that all utility parameters associated with the fishing regulations variables are jointly zero. In addition, we found in section S1 3.a that only 57% of respondents somewhat or strongly agree with the statement that “even if it costs us more money, we should try for a quick change rather than a slow change in Oregon Coast Coho salmon”, while 40% responded to the same statement with “neither agree or disagree” or somewhat or strongly disagree. Therefore, we suspect to find potential non-attendance with the fishing regulations parameters and the rate of return in returning fish parameters. We explore attribute non-attendance by estimating a latent class-RPL model as developed in Hess et al. (2013) where we estimate the probability of non-attendance for each of the five attributes in our choice questions while assuming independence of the probabilities of non-attendance of all attributes. Parameter estimates are presented in Table F. Included in the latent class-RPL estimates are Hess et al.’s attribute non-attendance (A-NA) parameters which are used to define probabilities of non-attendance classes as exp(A-NA parameter)/[1+exp(A-NA parameter)].

We use the attribute non-attendance parameters to estimate the probabilities of attribute non-attendance for each of the five attributes and present results in Table G. We find no evidence of attribute non-attendance for the price and the recovered attribute (5% level). We find that approximately 17% of the respondents did not attend to the fish return attribute, 43% did not attend to the rate attribute (dummy variable for quick), and 93% did not attend to the fishing regulation attributes. The larger estimated probabilities of attribute non-attendance for the rate and fishing regulations attributes are consistent with the earlier analyses suggesting that fewer respondents had strong preferences over these attributes.

We also estimate household WTP for the OC Coho salmon conservation scenarios for the latent class – RPL model and compare them to our preferred model in Table H. Despite the somewhat high estimated probabilities of attribute non-attendance for the rate of returning fish (quick) and fishing regulations in Table G, results in Table H suggest very similar estimated mean WTP in the latent class – RPL model and in the preferred model. Similar to Hess et al. (2013), we interpret these similarities in estimated mean WTP to imply that by explicitly modeling preference heterogeneity with random parameters in our preferred model, we are adequately able to account for the fact that some portion of the sample either does not attend to or has zero marginal utility over some of the attributes.

*S3.f Analysis of the role of stratification in the sample*

Our survey was mailed out with a stratified random sampling approach where residents of the state of Oregon were more likely to receive the survey than residents of Idaho, Washington, or northern California. In particular, we sent 2,500 surveys to a random sample of Oregon residents and 2,500 surveys to a random sample of non-Oregon residents. Table I presents the observed number of choice question responses by region, and shows that the higher percentage of responses from Oregon (58%) does not reflect Oregon’s share of the total population of households (17%). As such, we calculate weights by dividing the population percentage of the total population by the sample percentage of the population, and then weight the sample estimated log-likelihood function by the weights. This weighting ensures that our weighted number of choice question observations equals the sample number of choice question observations (2,734).

Table J compares unweighted results from our preferred model with results from estimating the same model with the weights from Table I. The weighting gives more weight to non-Oregon residents and less to Oregon residents. If there were systematic differences in the responses between Oregon and non-Oregon residents, then we would expect to see differences in the estimation results. Instead, Table J shows very similar estimation results across the weighted and unweighted samples.

Table K compares WTP estimates across the unweighted and weighted models, and estimated results are very similar. Using the weighted model, we would fail to reject the null that the mean WTP estimates are the same as the point estimates from the unweighted model (5% level). As such, we keep the unweighted model results as our preferred results.

*S3 g. Value Aggregation and Analysis of Sample Selection Bias*

The household WTP estimates above can be scaled up to consider the non-market values for the broader population of close to ten million households in our study region of the Pacific Northwest. In order to scale up our WTP estimates, results in Table 2 from the main text reveal that while our sample is representative of the broader Pacific Northwest population in terms of median household income, our sample is more educated, older, and more likely to be male and white than the population. In this section we analyze biases that arise from the fact that of the respondents who received a delivered survey, we had 21% respond and 79% that did not respond.

First, to understand any biases arising from our sample being non-representative in observable demographic characteristics, we consider whether the primary utility parameters systematically vary with education, age, gender, and race. Table L presents parameter estimates for the primary model which include an interaction between a dummy variable for whether the respondent has a 4-year college degree and the key utility parameters representing the recovery status of OC Coho ($\beta_{i2}$), the final number of returning fish ($\beta_{i3}$), and whether the number of returning fish increases quickly ($\beta_{i4}$). Since these are random parameters for individual respondent *i*, we interacted the means of the random parameters with our demographic variables. A likelihood ratio test for whether the three interaction parameters are jointly zero provides a test statistic of 18.52 with a corresponding p-value of 0.0003. Thus, we reject the null hypothesis of no significant interaction between the utility parameters for those with a 4-year college degree. In contrast, similar likelihood ratio tests for interactions between the utility parameters and a dummy variable for male, a dummy variable for age 65+, and a dummy variable for non-white all finds no evidence of a significant interaction between the utility parameters and these demographic variables. The p-values from each test are presented in Table 2 of the main text.

Second, we analyze sample selection based on unobservable characteristics. While there are no formal sample selection corrections for non-linear Logit models like ours, we instead follow Cameron and DeShazo (2013) and Kolstoe and Cameron (2017) and explicitly model the propensity of individuals to respond to our survey as a binary choice econometric model, and then estimate possible links between estimated response propensities and preferences for OC Coho conservation. We estimate drivers of whether or not individuals responded to our survey by using a rich set of observable information for both our respondents and non-respondents. Independent variables include demographic data for the census tracts or county where each individual lives, state dummy variables, and information about the mail delivery for each household. Estimation results are presented in Table M and show that individuals in counties that voted for the Democratic candidate (Clinton) in the 2016 presidential election were more likely to respond to our survey, California residents were less likely and Idaho residents were more likely to respond, and individuals with neighborhood delivery and collection box units (NDCBUs) were less likely to respond. No other parameters were significantly different from zero at any reasonable confidence level. People with NDCBU mail delivery likely faced higher costs of returning our surveys since such collection box units typically feature outgoing mail slots that are likely too small to fit our returned large-packet mail surveys. The importance of the NDCBUs in influencing survey response is that this variable provides us with an exclusion restriction that is typically necessary for tests of sample selection – a variable that influences selection into the survey, but should be uncorrelated with preferences for OC Coho. The mean probability to respond to the survey is 0.215. We then determined the difference between each individual’s estimated probability of responding and the mean of 0.215, *pdiff*, and included an interaction between *pdiff* and the key utility parameters representing the recovery status of OC Coho ($\beta_{i2}$), the final number of returning fish ($\beta_{i3}$), and whether the number of returning fish increases quickly ($\beta_{i4}$). Similar to the demographic information, we interacted the means of the random parameters with *pdiff*. Estimation results are presented in Table N. A likelihood ratio test for whether the three interaction parameters with *pdiff* are jointly zero provides a test statistic of 2.08 with a corresponding p-value of 0.56. Thus, we fail to reject the null hypothesis of no sample selection bias based on unobserved preferences for OC Coho conservation.

We conclude that value aggregation must account for differences between population and sample percentages of people with 4-year college degrees, but not age, gender, or race. We also find no evidence of sample selection based on unobserved preferences. Parameter estimates in Table L – Main Results with Interaction of College Degree Dummy – are used to generate mean WTP that is representative of the broader population. We generate representative WTP by substituting the population percentage of people with a 4-year college degree in decimals (0.358) in for the dummy variable of whether people have a college degree, and simulate mean WTP in the same way as in section 3c. Household level mean WTP estimates plus confidence intervals are presented in Table O.

We present two approaches to aggregation. First, we implement the so-called lower-bound approach (Loomis 1987), in which the sample WTP estimate is multiplied by a portion of the population while the remainder of the population is assumed to have a WTP of zero. The common practice for implementing the lower bound approach is to use the survey response rate as the portion of the population for which WTP is non-zero, while the remainder of the population is assumed to have zero WTP. We use the lower bound approach to generate an estimate of aggregate value, differentiating the response rate by Oregon (25.4%) and non-Oregon (17.8%) households. Our sample WTP estimates are then multiplied by 398,889 Oregon households (0.254×1,570,430 households), and by 1,395,098 non-Oregon Pacific Northwest households (0.178×7,837,629 households). Aggregate values of the primary model are presented in Figure E and in Table 3 of the main text. Second, our upper-bound approach uses the population-adjusted mean WTP estimate multiplied by the total population (9,408,059 households) to obtain the population level benefits. The last two columns in Table 3 in the main text show the results from the two approaches to value aggregation.

Finally, Table P presents population benefit estimates for changes in individual attributes of OC Coho salmon conservation using both the lower bound and upper bound approaches to value aggregation.

**S1 TEXT FIGURES**

**Figure A.** Example choice question

|  | **Results in 50 years** | | | |
| --- | --- | --- | --- | --- |
|  | **Status Quo** | | **Alternative A** | **Alternative B** |
| **Population Status** | Threatened | | Recovered | Recovered |
| **Population Size** | 150,000 fish | | 525,000 fish | 375,000 fish |
| **Population**  **over Time** | No Change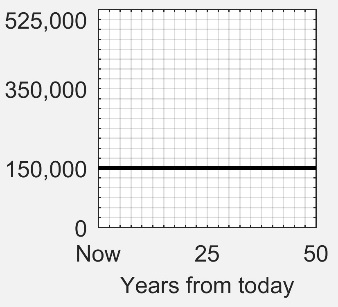 | | Slow Change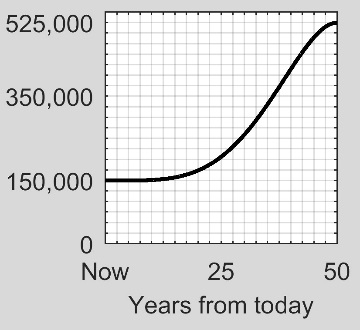 | Quick Change  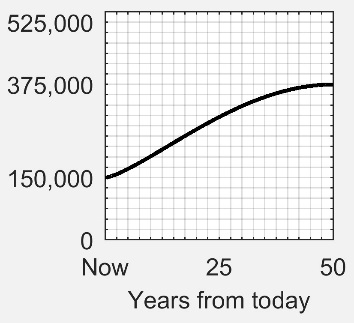 |
| **Recreational Fishing**  (# of fish that can be kept) | Periodically  Closed  5 fish/year | | Open Every  Year  10 fish/year | Open Every  Year  5 fish/year |
| **Added cost to your household each year** for 10 years | $0 | | $100/year | $350/year |
| **Which alternative do you prefer?**  ***(Choose One)*** | **⃝_1_** | **⃝_2_** | | **⃝_3_** |
|  | **Status Quo** | **Alternative A** | | **Alternative B** |

Note: This is one of 60 unique choice questions used in the design, where the levels of attributes vary across the choice questions

**Figure B.** Qualitative responses to auxiliary questions

**Figure C.** Stated Respondent Discount Rate (Mean = 21% for those ≤60%)

**Figure D.** Responses to auxiliary questions used to identify potential protest responses

**Figure E.** Lower bound aggregate annual non-market values of Oregon Coast Coho salmon conservation scenarios for the Pacific Northwest

**Figure F.** Upper bound aggregate annual non-market values of Oregon Coast Coho salmon conservation scenarios for the Pacific Northwest


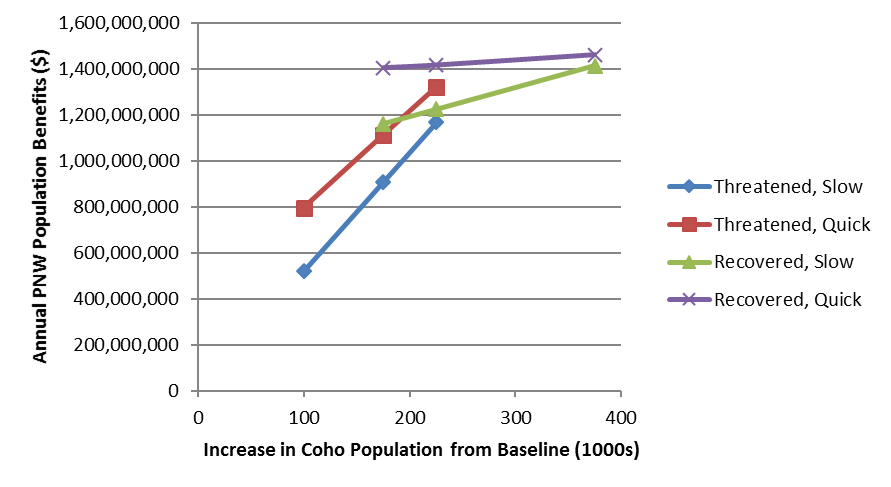


**SI TEXT Tables**

**Table A. Rules governing permissible combinations of non-cost attribute levels**

| **Attribute** | **Attribute Code** | **ESA Status** | **Number of returning fish** | **Rate** | **Fishing regulations** |
| --- | --- | --- | --- | --- | --- |
| ESA Status | 1 | - | 1,2,3,4 | 1,2,3 | 1,2 |
|  | 2 | - | 3,4,5 | 2,3 | 2,3 |
| Number of returning fish | 1 | 1 | - | 1 | 1 |
|  | 2 | 1 | - | 2,3 | 1,2 |
|  | 3 | 1,2 | - | 2,3 | 1,2,3 |
|  | 4 | 1,2 | - | 2,3 | 2,3 |
|  | 5 | 2 | - | 2,3 | 2,3 |
| Number of returning fish over time (Rate) | 1 | 1 | 1 | - | 1 |
|  | 2 | 1,2 | 2,3,4,5 | - | 1,2,3 |
|  | 3 | 1,2 | 2,3,4,5 | - | 1,2,3 |
| Fishing regulations | 1 | 1 | 1,2,3 | 1,2,3 | - |
|  | 2 | 1,2 | 2,3,4,5 | 2,3 | - |
|  | 3 | 2 | 3,4,5 | 2,3 | - |

Note: Numbers are attribute codes from Figure 2 in main text.

**Table B. Estimated Annual Mean Household Willingness-to-Pay for Oregon Coast Coho salmon conservation scenarios**

| ESA Status | Change in number of returning fish (1000s fish) | Rate of fish return | Simulated Mean WTP ($) | 95% CI ($) | |
| --- | --- | --- | --- | --- | --- |
| Threatened | 100 | Slow | 59.75 | 36.66 | 82.84 |
|  | 175 |  | 104.57 | 64.16 | 144.98 |
|  | 225 |  | 134.45 | 82.49 | 186.40 |
|  | 100 | Quick | 93.69 | 55.32 | 132.05 |
|  | 175 |  | 130.65 | 82.83 | 178.47 |
|  | 225 |  | 154.99 | 99.40 | 210.58 |
| Recovered | 175 | Slow | 135.19 | 83.41 | 186.96 |
|  | 225 |  | 144.02 | 93.69 | 194.35 |
|  | 375 |  | 170.58 | 119.36 | 221.81 |
|  | 175 | Quick | 164.88 | 107.36 | 222.40 |
|  | 225 |  | 168.29 | 116.13 | 220.46 |
|  | 375 |  | 179.19 | 131.91 | 226.46 |

**Table C. Estimated Annual Mean Household Willingness-to-Pay for changes in individual attributes of Oregon Coast Coho salmon conservation**

| WTP for: | Evaluated at: | | | Estimates | | |
| --- | --- | --- | --- | --- | --- | --- |
|  | *ESA Status* | *Change in number of returning fish (1000s)* | *Rate of fish return* | *WTP* | *95% CI* | |
| 100,000 more returning fish | Threatened | Any | Slow | 59.75 | 36.66 | 82.84 |
|  |  |  | Quick | 49.28 | 27.95 | 70.62 |
|  | Recovered |  | Slow | 11.77 | 1.95 | 21.60 |
|  |  |  | Quick | 4.55 | -10.89 | 19.99 |
| Quick | Threatened | 100 | Slow | 33.93 | 13.49 | 54.37 |
|  |  | 175 |  | 26.08 | 11.15 | 41.01 |
|  |  | 225 |  | 20.54 | 4.18 | 36.90 |
|  | Recovered | 175 |  | 29.69 | 15.48 | 43.91 |
|  |  | 225 |  | 24.28 | 8.82 | 39.74 |
|  |  | 375 |  | 8.60 | -22.62 | 39.82 |
| Recovered | Threatened | 175 | Slow | 30.62 | 0.00 | 61.23 |
|  |  | 225 |  | 9.57 | -19.39 | 38.53 |
|  |  | 175 | Quick | 34.23 | 3.76 | 64.70 |
|  |  | 225 |  | 13.31 | -15.29 | 41.90 |

**Table D. Random Parameters Logit (RPL) Estimation Results for Preferred Model using Full Sample and Sample that drops possible “Protest Respondents”**

|  |  | Full Sample | | | No “Protest Respondents” | | |
| --- | --- | --- | --- | --- | --- | --- | --- |
|  |  | Parameter | Stand. error | t-stat | Parameter | Stand. error | t-stat |
| Status quo | Alt. Spec. Constant | -11.81* | 1.68 | -7.04 | -1.24* | 0.24 | -5.13 |
|  |  |  |  |  |  |  |  |
| Estimated parameter means of conservation alternatives | log(Price) | 0.85* | 0.14 | 6.26 | -0.33* | 0.08 | -3.99 |
|  | Recovered | 2.58* | 0.61 | 4.24 | 1.08* | 0.30 | 3.53 |
|  | ReturningFish | 1.70* | 0.31 | 5.53 | 0.59* | 0.15 | 3.91 |
|  | Quick | 1.18* | 0.37 | 3.18 | 0.48* | 0.20 | 2.45 |
|  | Fishing1 | -0.25 | 0.16 | -1.53 | -0.28* | 0.10 | -2.90 |
|  | Fishing2 | -0.34 | 0.27 | -1.26 | -0.59* | 0.15 | -3.94 |
|  | Recovered*  ReturningFish | -0.88* | 0.26 | -3.35 | -0.32* | 0.14 | -2.26 |
|  | Quick*  ReturningFish | -0.22 | 0.17 | -1.29 | -0.16 | 0.08 | -1.97 |
|  |  |  |  |  |  |  |  |
| Estimated parameter standard deviations of conservation alternatives | St. Dev.(Price) | 1.66* | 0.07 | 22.72 | 0.43 | 0.22 | 1.91 |
|  | St. Dev.(Recovered) | 1.17 | 1.00 | 1.17 | 0.66* | 0.33 | 2.02 |
|  | St. Dev. (ReturningFish) | 0.88* | 0.21 | 4.26 | 0.43* | 0.08 | 5.59 |
|  | St. Dev. (Quick) | 0.78* | 0.32 | 2.43 | 0.21 | 0.25 | 0.82 |
|  | St. Dev. (Fishing1) | 1.04* | 0.44 | 2.34 | 0.06 | 0.42 | 0.15 |
|  | St. Dev. (Fishing2) | 0.28 | 0.93 | 0.30 | 0.03 | 0.37 | 0.09 |
|  | St. Dev. (Recovered*  ReturningFish) | 0.61* | 0.21 | 2.95 | 0.04 | 0.19 | 0.20 |
|  | St. Dev. (Quick*  ReturningFish) | 0.53* | 0.19 | 2.85 | 0.01 | 0.32 | 0.02 |
|  | St. Dev. (ASC) | 10.81 | 1.42 | 7.62 | 0.01 | 0.39 | 0.02 |
|  |  |  |  |  |  |  |  |
|  | Log-likelihood | -2016.16 |  |  | -1993.05 |  |  |
|  | N | 2,734 |  |  | 2,216 |  |  |

Note: *Significant at the 1% level

**Table E. Estimated Annual Mean Household Willingness-to-Pay for Oregon Coast Coho salmon conservation scenarios – Full sample compared to sample with potential protests dropped**

|  | |  | Full Sample | | | No Protest Sample | | |
| --- | --- | --- | --- | --- | --- | --- | --- | --- |
| Status | Change in Fish Return (1000s) | Rate of return | Mean WTP ($) | 95% CI ($) | | Mean WTP ($) | 95% CI ($) | |
| Threatened | 100 | Slow | 59.75 | 36.66 | 82.84 | 75.62 | 38.60 | 112.65 |
|  | 175 |  | 104.57 | 64.16 | 144.98 | 132.34 | 67.55 | 197.13 |
|  | 225 |  | 134.45 | 82.49 | 186.40 | 170.15 | 86.85 | 253.46 |
|  | 100 | Quick | 93.69 | 55.32 | 132.05 | 116.96 | 56.44 | 177.49 |
|  | 175 |  | 130.65 | 82.83 | 178.47 | 156.86 | 79.80 | 233.91 |
|  | 225 |  | 154.99 | 99.40 | 210.58 | 183.54 | 93.26 | 273.82 |
| Recovered | 175 | Slow | 135.19 | 83.41 | 186.96 | 195.58 | 123.88 | 267.28 |
|  | 225 |  | 144.02 | 93.69 | 194.35 | 211.64 | 139.83 | 283.45 |
|  | 375 |  | 170.58 | 119.36 | 221.81 | 260.95 | 182.40 | 339.50 |
|  | 175 | Quick | 164.88 | 107.36 | 222.40 | 220.51 | 138.80 | 302.22 |
|  | 225 |  | 168.29 | 116.13 | 220.46 | 225.38 | 149.42 | 301.35 |
|  | 375 |  | 179.19 | 131.91 | 226.46 | 241.52 | 172.14 | 310.89 |

**Table F. Random Parameters Logit (RPL) Estimation Results for Preferred Model compared to a Latent-Class-RPL Attribute Non-Attendance model from Hess et al. (2013)**

|  |  | Full Sample | | | Latent Class-RPL | | |
| --- | --- | --- | --- | --- | --- | --- | --- |
|  |  | Parameter | Stand. error | t-stat | Parameter | Stand. error | t-stat |
| Status quo | Alt. Spec. Constant | -11.81* | 1.68 | -7.04 | -19.67* | 4.22 | -4.66 |
| Estimated parameter means of conserve. alternatives |  | 0.85* | 0.14 | 6.26 | 1.34* | 0.19 | 7.09 |
|  | log(Price) | 2.58* | 0.61 | 4.24 | 3.68* | 0.87 | 4.25 |
|  | Recovered | 1.70* | 0.31 | 5.53 | 2.50* | 0.51 | 4.94 |
|  | ReturningFish | 1.18* | 0.37 | 3.18 | 2.74* | 0.76 | 3.63 |
|  | Quick | -0.25 | 0.16 | -1.53 | -10.44* | 3.99 | -2.62 |
|  | Fishing1 | -0.34 | 0.27 | -1.26 | -21.65* | 6.07 | -3.57 |
|  | Fishing2 | -0.88* | 0.26 | -3.35 | -1.24* | 0.35 | -3.49 |
|  | Recovered*  ReturningFish | -0.22 | 0.17 | -1.29 | -0.44* | 0.22 | -2.02 |
| Estimated parameter standard deviations of conserve. alternatives | Quick*  ReturningFish | 1.66* | 0.07 | 22.72 | 1.59* | 0.07 | 22.68 |
|  |  | 1.17 | 1.00 | 1.17 | 2.50* | 0.85 | 2.95 |
|  | St. Dev.(Price) | 0.88* | 0.21 | 4.26 | 0.79* | 0.28 | 2.83 |
|  | St. Dev.(Recovered) | 0.78* | 0.32 | 2.43 | 1.06 | 0.57 | 1.87 |
|  | St. Dev. (ReturningFish) | 1.04* | 0.44 | 2.34 | 23.94* | 6.62 | 3.62 |
|  | St. Dev. (Quick) | 0.28 | 0.93 | 0.30 | 1.13 | 0.79 | 1.43 |
|  | St. Dev. (Fishing1) | 0.61* | 0.21 | 2.95 | 0.70* | 0.26 | 2.72 |
|  | St. Dev. (Fishing2) | 0.53* | 0.19 | 2.85 | 0.79 | 0.54 | 1.48 |
|  | St. Dev. (Recovered*  ReturningFish) | 10.81* | 1.42 | 7.62 | 17.79* | 3.60 | 4.94 |
|  |  |  |  |  |  |  |  |
|  | Price A-NA parameter |  |  |  | -2.79* | 0.75 | -3.71 |
|  | Recovered A-NA parameter |  |  |  | -13.43 | 316.57 | -0.04 |
|  | ReturningFish A-NA parameter |  |  |  | -1.61* | 0.45 | -3.61 |
|  | Quick A-NA parameter |  |  |  | -0.28 | 0.65 | -0.43 |
|  | Fishing A-NA parameter |  |  |  | 2.62* | 0.34 | 7.70 |
|  | Log-likelihood | -2016.16 |  |  | -1990.87 |  |  |
|  | N | 2,734 |  |  | 2,734 |  |  |

Note: *Significant at the 1% level

**Table G. Estimated probabilities of attribute non-attendance**

|  | Probability A-NA | Stand. Dev. |
| --- | --- | --- |
| Price | 0.06 | 0.05 |
| Recovered | 0.00 | 0.50 |
| Pop | 0.17* | 0.06 |
| Quick | 0.43* | 0.15 |
| Fishing | 0.93* | 0.02 |

Note: * Significant at the 1% level

**Table H. Estimated Annual Mean Household Willingness-to-Pay for Oregon Coast Coho salmon conservation scenarios – Preferred model compared to Latent Class – RPL Attribute Non-Attendance Model**

|  | |  | Preferred Model | | | Latent Class – RPL Model | | |
| --- | --- | --- | --- | --- | --- | --- | --- | --- |
| Status | Change in Fish Returns (1000s) | Rate of return | Mean WTP ($) | 95% CI ($) | | Mean WTP ($) | 95% CI ($) | |
| Threatened | 100 | Slow | 59.75 | 36.66 | 82.84 | 58.81 | 38.85 | 78.77 |
|  | 175 |  | 104.57 | 64.16 | 144.98 | 102.92 | 67.99 | 137.84 |
|  | 225 |  | 134.45 | 82.49 | 186.40 | 132.32 | 87.42 | 177.23 |
|  | 100 | Quick | 93.69 | 55.32 | 132.05 | 85.48 | 27.99 | 142.97 |
|  | 175 |  | 130.65 | 82.83 | 178.47 | 118.85 | 49.21 | 188.48 |
|  | 225 |  | 154.99 | 99.40 | 210.58 | 141.31 | 63.68 | 218.93 |
| Recovered | 175 | Slow | 135.19 | 83.41 | 186.96 | 103.09 | 53.26 | 152.91 |
|  | 225 |  | 144.02 | 93.69 | 194.35 | 116.61 | 63.54 | 169.67 |
|  | 375 |  | 170.58 | 119.36 | 221.81 | 156.02 | 93.36 | 218.68 |
|  | 175 | Quick | 164.88 | 107.36 | 222.40 | 149.06 | 76.14 | 221.97 |
|  | 225 |  | 168.29 | 116.13 | 220.46 | 156.39 | 83.00 | 229.77 |
|  | 375 |  | 179.19 | 131.91 | 226.46 | 176.18 | 98.83 | 253.53 |

**Table I. Calculation of weights to represent stratified random sample**

|  | Observed Choice question Responses | Number of households | Population  percentage of total population | Sample percentage of total population | Weight |
| --- | --- | --- | --- | --- | --- |
| Oregon | 1,574 | 1,570,430 | 0.17 | 0.58 | 0.29 |
| Non-Oregon | 1,160 | 7,837,629 | 0.83 | 0.42 | 1.96 |

**Table J. Random Parameters Logit (RPL) Estimation Results for Preferred Model using unweighted full sample and a sample weighted to represent stratified random sampling**

|  |  | Unweighted | | | Weighted | | |
| --- | --- | --- | --- | --- | --- | --- | --- |
|  |  | Parameter | Stand. error | t-stat | Parameter | Stand. error | t-stat |
| Status quo | Alt. Spec. Constant | -11.81* | 1.68 | -7.04 | -12.67* | 2.13 | -5.94 |
|  |  |  |  |  |  |  |  |
| Estimated parameter means of conserv. alternatives | log(Price) | 0.85* | 0.14 | 6.26 | 0.83* | 0.15 | 5.67 |
|  | Recovered | 2.58* | 0.61 | 4.24 | 2.41* | 0.60 | 4.03 |
|  | ReturningFish | 1.70* | 0.31 | 5.53 | 1.64* | 0.31 | 5.33 |
|  | Quick | 1.18* | 0.37 | 3.18 | 1.07* | 0.36 | 2.96 |
|  | Fishing1 | -0.25 | 0.16 | -1.53 | -0.23 | 0.16 | -1.48 |
|  | Fishing2 | -0.34 | 0.27 | -1.26 | -0.28 | 0.26 | -1.08 |
|  | Recovered*  ReturningFish | -0.88* | 0.26 | -3.35 | -0.83* | 0.26 | -3.20 |
|  | Quick*  ReturningFish | -0.22 | 0.17 | -1.29 | -0.16 | 0.16 | -1.02 |
|  |  |  |  |  |  |  |  |
| Estimated parameter standard deviations of conserv. alternatives | St. Dev.(Price) | 1.66* | 0.07 | 22.72 | 1.71* | 0.07 | 24.02 |
|  | St. Dev.(Recovered) | 1.17 | 1.00 | 1.17 | 1.98* | 0.61 | 3.24 |
|  | St. Dev. (ReturningFish) | 0.88* | 0.21 | 4.26 | 0.84* | 0.28 | 2.95 |
|  | St. Dev. (Quick) | 0.78* | 0.32 | 2.43 | 0.81* | 0.33 | 2.45 |
|  | St. Dev. (Fishing1) | 1.04* | 0.44 | 2.34 | 0.80 | 0.51 | 1.58 |
|  | St. Dev. (Fishing2) | 0.28 | 0.93 | 0.30 | 0.17 | 0.74 | 0.23 |
|  | St. Dev. (Recovered*  ReturningFish) | 0.61* | 0.21 | 2.95 | 0.37 | 0.30 | 1.24 |
|  | St. Dev. (Quick*  ReturningFish) | 0.53* | 0.19 | 2.85 | 0.45* | 0.21 | 2.12 |
|  | St. Dev. (ASC) | 10.81 | 1.42 | 7.62 | 11.86* | 2.07 | 5.73 |
|  |  |  |  |  |  |  |  |
|  | Log-likelihood | -2016.16 |  |  | -3183.79 |  |  |
|  | N | 2,734 |  |  | 2,734 |  |  |

Note: *Significant at the 1% level

**Table K. Estimated Annual Mean Household Willingness-to-Pay (WTP) for Oregon Coast Coho salmon conservation scenarios – Preferred model compared to Weighted Model**

|  | |  | Unweighted Model | | | Weighted Model | | |
| --- | --- | --- | --- | --- | --- | --- | --- | --- |
| Status | Change in Fish Returns (1000s) | Rate | Mean WTP ($) | 95% CI ($) | | Mean WTP ($) | 95% CI ($) | |
| Threatened | 100 | Slow | 59.75 | 36.66 | 82.84 | 58.86 | 35.16 | 82.56 |
|  | 175 |  | 104.57 | 64.16 | 144.98 | 103.00 | 61.52 | 144.48 |
|  | 225 |  | 134.45 | 82.49 | 186.40 | 132.43 | 79.10 | 185.75 |
|  | 100 | Quick | 93.69 | 55.32 | 132.05 | 91.34 | 51.54 | 131.14 |
|  | 175 |  | 130.65 | 82.83 | 178.47 | 130.27 | 80.32 | 180.21 |
|  | 225 |  | 154.99 | 99.40 | 210.58 | 155.62 | 97.39 | 213.84 |
| Recovered | 175 | Slow | 135.19 | 83.41 | 186.96 | 124.78 | 74.15 | 175.41 |
|  | 225 |  | 144.02 | 93.69 | 194.35 | 136.14 | 85.92 | 186.37 |
|  | 375 |  | 170.58 | 119.36 | 221.81 | 168.46 | 115.24 | 221.68 |
|  | 175 | Quick | 164.88 | 107.36 | 222.40 | 155.55 | 97.41 | 213.69 |
|  | 225 |  | 168.29 | 116.13 | 220.46 | 162.09 | 108.88 | 215.30 |
|  | 375 |  | 179.19 | 131.91 | 226.46 | 181.00 | 132.24 | 229.76 |

**Table L. Random Parameters Logit (RPL) Estimation Results for Primary Model compared to a model that includes an interaction with a dummy variable indicating whether the respondent had a 4-year college degree**

|  |  | Main Results | | | Main Results with Interaction of College Degree Dummy | | |
| --- | --- | --- | --- | --- | --- | --- | --- |
|  |  | Parameter | Stand. error | t-stat | Parameter | Stand. error | t-stat |
| Status quo | Alt. Spec. Constant | -11.81* | 1.68 | -7.04 | -11.74* | 1.91 | -6.14 |
| Estimated parameter means of conservation alternatives | log(Price) | 0.85* | 0.14 | 6.26 | 0.86* | 0.14 | 6.17 |
|  | Recovered | 2.58* | 0.61 | 4.24 | 2.50* | 0.63 | 3.95 |
|  | ReturningFish | 1.70* | 0.31 | 5.53 | 1.31* | 0.31 | 4.29 |
|  | Quick | 1.18* | 0.37 | 3.18 | 0.91* | 0.38 | 2.41 |
|  | Fishing1 | -0.25 | 0.16 | -1.53 | -0.25 | 0.16 | -1.54 |
|  | Fishing2 | -0.34 | 0.27 | -1.26 | -0.30 | 0.26 | -1.13 |
|  | Rec*Pop | -0.88* | 0.26 | -3.35 | -0.88* | 0.26 | -3.38 |
|  | Quick*ReturnFish | -0.22 | 0.17 | -1.29 | -0.21 | 0.16 | -1.26 |
| Estimated parameter standard deviations of conservation alternatives | St. Dev.(Price) | 1.66* | 0.07 | 22.72 | 1.66* | 0.08 | 22.08 |
|  | St. Dev.(Recovered) | 1.17 | 1.00 | 1.17 | 1.32 | 0.74 | 1.77 |
|  | St. Dev. (ReturningFish) | 0.88* | 0.21 | 4.26 | 0.79* | 0.19 | 4.09 |
|  | St. Dev. (Quick) | 0.78* | 0.32 | 2.43 | 0.79* | 0.33 | 2.42 |
|  | St. Dev. (Fishing1) | 1.04* | 0.44 | 2.34 | 0.96* | 0.43 | 2.24 |
|  | St. Dev. (Fishing2) | 0.28 | 0.93 | 0.30 | 0.26 | 0.78 | 0.33 |
|  | St. Dev. (Recover*  ReturningFish) | 0.61* | 0.21 | 2.95 | 0.62* | 0.22 | 2.78 |
|  | St. Dev. (Quick*  ReturningFish) | 0.53* | 0.19 | 2.85 | 0.51* | 0.19 | 2.75 |
|  | St. Dev. (ASC) | 10.81* | 1.42 | 7.62 | 10.86* | 1.71 | 6.35 |
|  |  |  |  |  |  |  |  |
| Estimated parameters for interactions with College | Recovered*College |  |  |  | 0.10 | 0.39 | 0.27 |
|  | ReturningFish*College |  |  |  | 0.73* | 0.20 | 3.59 |
|  | Quick*College |  |  |  | 0.49 | 0.26 | 1.89 |
|  |  |  |  |  |  |  |  |
|  |  |  |  |  |  |  |  |
|  | Log-likelihood | -2016.16 |  |  | -2006.9 |  |  |
|  | N | 2,734 |  |  | 2,734 |  |  |

Note: *Significant at the 1% level

**Table M. Binary logit model of propensity to respond to survey**

|  |  | Dependent Variable = 1 if Respond to Survey | | |
| --- | --- | --- | --- | --- |
|  |  | Parameter | Stand. error | t-stat |
| Census Tract variables | Percent Hispanic | -0.02 | 0.01 | -1.58 |
|  | Percent African Am | -0.03 | 0.02 | -1.38 |
|  | Percent Asian | -0.02 | 0.02 | -1.29 |
|  | Percent White | -0.004 | 0.02 | -0.23 |
|  | Median Income | 1.36E-06 | 2.10E-06 | 0.65 |
| County variable | Percent Democratic Vote in 2016 Presidential | 0.01* | 0.004 | 2.59 |
|  |  |  |  |  |
| Individual variables | California (yes=1) | -0.55* | 0.13 | -4.09 |
|  | Washington (yes=1) | -0.12 | 0.10 | -1.14 |
|  | Idaho (yes=1) | 0.42** | 0.20 | 2.07 |
|  | Mail delivery - curb (yes=1) | -0.03 | 0.13 | -0.23 |
|  | Mail deilvery - NDCBU (yes=1) | -0.26** | 0.15 | -1.78 |
|  | Mail delivery - central (yes=1) | -0.28 | 0.25 | -1.13 |
|  | High rise building (yes=1) | 0.25 | 0.19 | 1.32 |
|  | Intercept | -1.11 | 1.57 | -0.70 |
|  | Log-likelihood | -2314.1 |  |  |
|  | N | 4,381 |  |  |

Note: *Significant at the 1% level; **Significant at the 10% level

**Table N. Random Parameters Logit (RPL) Estimation Results for including interactions between preference parameters and estimated propensity to respond to the survey**

|  |  | With Interaction of College Degree Dummy | | | With Interaction of Sample Selection Probability Difference (*pdiff*) | | |
| --- | --- | --- | --- | --- | --- | --- | --- |
|  |  | Parameter | Stand. error | t-stat | Parameter | Stand. error | t-stat |
| Status quo | Alt. Spec. Constant | -11.74* | 1.91 | -6.14 | -11.65* | 1.92 | -6.07 |
| Estimated parameter means of conservation alternatives | log(Price) | 0.86* | 0.14 | 6.17 | 0.86* | 0.14 | 6.15 |
|  | Recovered | 2.50* | 0.63 | 3.95 | 2.41* | 0.64 | 3.78 |
|  | ReturningFish | 1.31* | 0.31 | 4.29 | 1.30* | 0.31 | 4.23 |
|  | Quick | 0.91* | 0.38 | 2.41 | 0.89* | 0.38 | 2.32 |
|  | Fishing1 | -0.25 | 0.16 | -1.54 | -0.25 | 0.16 | -1.55 |
|  | Fishing2 | -0.30 | 0.26 | -1.13 | -0.31 | 0.27 | -1.15 |
|  | Rec*Pop | -0.88* | 0.26 | -3.38 | -0.87* | 0.26 | -3.32 |
|  | Quick*ReturnFish | -0.21 | 0.16 | -1.26 | -0.21 | 0.17 | -1.28 |
| Estimated parameter standard deviations of conservation alternatives | St. Dev.(Price) | 1.66* | 0.08 | 22.08 | 1.66* | 0.08 | 21.85 |
|  | St. Dev.(Recovered) | 1.32 | 0.74 | 1.77 | 1.17 | 0.86 | 1.36 |
|  | St. Dev. (ReturningFish) | 0.79* | 0.19 | 4.09 | 0.79* | 0.19 | 4.09 |
|  | St. Dev. (Quick) | 0.79* | 0.33 | 2.42 | 0.81* | 0.33 | 2.46 |
|  | St. Dev. (Fishing1) | 0.96* | 0.43 | 2.24 | 1.02* | 0.43 | 2.39 |
|  | St. Dev. (Fishing2) | 0.26 | 0.78 | 0.33 | 0.26 | 0.77 | 0.34 |
|  | St. Dev. (Recover*  ReturningFish) | 0.62* | 0.22 | 2.78 | 0.65* | 0.24 | 2.71 |
|  | St. Dev. (Quick*  ReturningFish) | 0.51* | 0.19 | 2.75 | 0.51* | 0.19 | 2.74 |
|  | St. Dev. (ASC) | 10.86* | 1.71 | 6.35 | 10.74* | 1.75 | 6.12 |
|  |  |  |  |  |  |  |  |
| Interactions with College | Recovered*College | 0.10 | 0.39 | 0.27 | 0.09 | 0.39 | 0.24 |
|  | ReturningFish*College | 0.73* | 0.20 | 3.59 | 0.73* | 0.21 | 3.57 |
|  | Quick*College | 0.49 | 0.26 | 1.89 | 0.48 | 0.26 | 1.85 |
| Interactions with pdiff | Recovered*pdiff |  |  |  | 3.45 | 3.21 | 1.08 |
|  | ReturningFish*pdiff |  |  |  | -0.25 | 1.53 | -0.16 |
|  | Quick*pdiff |  |  |  | 1.91 | 2.02 | 0.94 |
|  | Log-likelihood | -2006.9 |  |  | -2005.86 |  |  |
|  | N | 2,734 |  |  | 2,734 |  |  |

Note: *Significant at the 1% level

**Table O. Estimated Annual Mean Household Willingness-to-Pay for Oregon Coast Coho Salmon Conservation Scenarios – Adjusted to Population of Pacific Northwest Residents**

| **ESA Status** | **Change in Number of Returning Fish (1000s fish)** | **Rate of Fish Return** | **Simulated Mean WTP ($)** | **95% CI ($)** | |
| --- | --- | --- | --- | --- | --- |
| Threatened | 100 | Slow | 55.13 | 32.48 | 79.4 |
|  | 175 |  | 96.47 | 56.85 | 138.95 |
|  | 225 |  | 124.04 | 73.09 | 178.64 |
|  | 100 | Quick | 84.62 | 47.32 | 124.61 |
|  | 175 |  | 118.29 | 71.97 | 168.51 |
|  | 225 |  | 140.43 | 87.11 | 199.57 |
| Recovered | 175 | Slow | 123.45 | 75.06 | 177.89 |
|  | 225 |  | 130.04 | 82.81 | 183.32 |
|  | 375 |  | 150.08 | 101.97 | 204.75 |
|  | 175 | Quick | 149.29 | 94.73 | 209.84 |
|  | 225 |  | 150.53 | 101.00 | 206.37 |
|  | 375 |  | 155.30 | 112.02 | 204.36 |

**Table P. Lower and Upper Bound estimated annual Pacific Northwest population benefits for changes in individual attributes of OC Coho salmon conservation**

| WTP for: | Evaluated at: | | | Estimates | | | |
| --- | --- | --- | --- | --- | --- | --- | --- |
|  | *ESA Status* | *Change in number of returning fish (1000s)* | *Rate of fish return* | *Sample mean hshld WTP ($)* | *Lower Bound Population Benefits ($)***=** *sample mean hshld WTP x 1,793,987 households* | *Pop-Adj mean hshld WTP ($)* | *Upper Bound Population Benefits ($)***=** *pop-adj mean hshld WTP x 9,408,059 households* |
| 100,000 more returning fish | Threat | Any | Slow | 59.75 | 107,198,154 | 55.13 | 518,652,033 |
|  |  |  | Quick | 49.28 | 88,415,755 | 44.89 | 422,323,276 |
|  | Recover |  | Slow | 11.77 | 21,122,716 | 8.79 | 82,719,985 |
|  |  |  | Quick | 4.55 | 8,166,634 | 1.65 | 15,512,257 |
| Quick | Threat | 100 | Slow | 33.93 | 60,872,680 | 29.49 | 277,476,812 |
|  |  | 175 |  | 26.08 | 46,785,881 | 21.81 | 205,230,242 |
|  |  | 225 |  | 20.54 | 36,853,613 | 16.39 | 154,234,242 |
|  | Recover | 175 |  | 29.69 | 53,270,545 | 25.84 | 243,124,305 |
|  |  | 225 |  | 24.28 | 43,553,484 | 20.48 | 192,718,510 |
|  |  | 375 |  | 8.60 | 15,432,382 | 5.22 | 49,103,903 |
| Recover | Threat | 175 | Slow | 30.62 | 54,926,497 | 26.97 | 253,780,242 |
|  |  | 225 |  | 9.57 | 17,169,457 | 6.00 | 56,494,214 |
|  |  | 175 | Quick | 34.23 | 61,411,161 | 31.00 | 291,674,302 |
|  |  | 225 |  | 13.31 | 23,869,328 | 10.10 | 94,978,482 |

**References for Supporting Information (S1 Text)**

The American Association for Public Opinion Research (AAPOR) (2016) *Standard Definitions: Final Dispositions of Case Codes and Outcome Rates for Surveys. 9th edition*. AAPOR.

Dillman, D.A., V. Lesser, R. Mason, J. Carlson, F. Willits, R. Robertson, and B. Burke (2007) “Personalization of Mail Surveys for General Public and Populations with a Group Identity: Results from Nine Studies.” *Rural Sociology* 72(4):632-646.

Dillman, D. A., J. D. Smyth and L. M. Christian (2014). *Internet, Phone, Mail, and Mixed-Mode Surveys: The Tailored Design Method.* 4^th^ edition. Hoboken, New Jersey, John Wiley and Sons.

Hess, S., A. Stathopoulos, D. Campbell, V. O’Neill, and S. Caussade (2013) “It’s not that I don’t care, I just don’t care very much: Confounding between attribute non-attendance and taste heterogeneity” *Transportation* 40: 583-607.

Huber, J., and K. Zwerina (1996) “The Importance of Utility Balance in Efficient Choice Designs” *Journal of Marketing Research* 33(3): 307-317.

Johnson, F.R., E. Lancsar, D. Marshall, V. Kilambi, A. Muhlbacher, D.A. Regier, B.W. Bresnahan, B. Kanninen, and J.F.P. Bridges (2013) “Constructing Experimental Designs for Discrete-Choice Experiments: Report of the ISPOR Conjoint Analysis Experimental Design Good Research Practices Task Force” *Value in Health* 16:3-13.

Johnston, R.J., Boyle, K.J., Adamowicz, W., Bennett, J., Brouwer, R., Cameron, T.A., Hanemann, W.M., Hanley, N., Ryan, M., Scarpa, R. and Tourangeau, R., 2017. Contemporary guidance for stated preference studies. *Journal of the Association of Environmental and Resource Economists*, *4*(2), pp.319-405.

Krinsky, I., Robb, A.L. 1986. On Approximating the Statistical Properties of Elasticities. *Review of Economic and Statistics* 68, 715-719.

Lew, D.K., D.F. Layton, R.D. Rowe RD (2010) Valuing Enhancements to Endangered Species Protection under Alternative Baseline Futures: The Case of the Steller Sea Lion. *Marine Resource Economics* 25:133-154.

Lew, D.K., and K. Wallmo (2011) External Tests of Embedding and Scope in Stated Preference Choice Experiments: An Application to Endangered Species Valuation. *Environmental and Resource Economics* 48(1):1-23.

Loomis, J.B. (1987) “Expanding Contingent Value Sample Estimates to Aggregate Benefit Estimates: Current Practices and Proposed Solutions.” *Land Economics* 63(4): 396-402.

Louviere, J.J., D.A. Hensher, J.D. Swait (2000) *Stated Choice Methods: Analysis and Application*. Cambridge University Press, Cambridge, UK.

MacDonald, D.H., M.D. Morrison, J.M. Rose, and K.J. Boyle (2011) Valuing a Multistate River: The Case of the River Murray. *Australian Journal of Agricultural and Resource Economics* 55:374-392.

Meyerhoff, J., and U. Liebe (2010) “Determinants of protest responses in environmental valuation: A meta-study” *Ecological Economics* 70: 366-374.

Mitchell, R.C., and R.T. Carson (1989) *Using Surveys to Value Public Goods: The Contingent Valuation Method*, Washington D.C.: Resources for the Future.

Scarpa, R., T.J. Gilbride, D. Campbell, and D.A. Hensher (2009) “Modelling attribute non-attendance in choice experiments for rural landscape valuation.” *European Review of Agricultural Economics* 36(2): 151-174.

Train, K.E. (2009) Discrete Choice Methods with Simulation, 2^nd^ edition. New York: Cambridge University Press.

Wakamatsu, M, KJ Shin, C Wilson, and S Managi (2018) Exploring a Gap between Australia and Japan in the Economic Valuation of Whale Conservation. *Ecological Economics* 146: 397-407.

Yin, X., Goudriaan, J.A.N., Lantinga, E.A., Vos, J.A.N. and H.J. Spiertz. (2003) A flexible sigmoid function of determinate growth. *Annals of botany* 91(3): 361-371.
